# Dissecting and Reconstructing Matrix in Malignant Mesothelioma Through Histocell-Histochemistry Gradients for Clinical Applications

**DOI:** 10.3389/fmed.2022.871202

**Published:** 2022-04-13

**Authors:** Marcelo Luiz Balancin, Camila Machado Baldavira, Tabatha Gutierrez Prieto, Juliana Machado-Rugolo, Cecília Farhat, Aline Kawassaki Assato, Ana Paula Pereira Velosa, Walcy Rosolia Teodoro, Alexandre Muxfeldt Ab'Saber, Teresa Yae Takagaki, Vera Luiza Capelozzi

**Affiliations:** ^1^Laboratory of Genomics and Histomorphometry, Department of Pathology, University of São Paulo Medical School (USP), São Paulo, Brazil; ^2^Health Technology Assessment Center (NATS), Clinical Hospital (HCFMB), Medical School of São Paulo State University (UNESP), Botucatu, Brazil; ^3^Rheumatology Division of the Hospital das Clinicas da Faculdade de Medicina da Universidade de São Paulo, FMUSP, São Paulo, Brazil; ^4^Division of Pneumology, Instituto do Coração (Incor), University of São Paulo Medical School (USP), São Paulo, Brazil

**Keywords:** malignant mesothelioma, Movat's pentachrome stain, Picrosirius, immunohistochemistry, prognosis

## Abstract

**Background:**

Malignant pleural mesotheliomas (MM) are known for their heterogenous histology and clinical behavior. MM histology reveals three major tumor cell populations: epithelioid, sarcomatoid, and biphasic. Using a dissecting approach, we showed that histochemical gradients help us better understand tumor heterogeneity and reconsider its histologic classifications. We also showed that this method to characterize MM tumor cell populations provides a better understanding of the underlying mechanisms for invasion and disease progression.

**Methods:**

In a cohort of 87 patients with surgically excised MM, we used hematoxylin and eosin to characterize tumor cell populations and Movat's pentachrome staining to dissect the ECM matrisome. Next, we developed a computerized semi-assisted protocol to quantify and reconstruct the ECM in 3D and examined the clinical association between the matricellular factors and patient outcome.

**Results:**

Epithelioid cells had a higher matrix composition of elastin and fibrin, whereas, in the sarcomatoid type, hyaluronic acid and total collagen were most prevalent. The 3D reconstruction exposed the collagen I and III that form channels surrounding the neoplastic cell blocks. The estimated volume of the two collagen fractions was 14% of the total volume, consistent with the median estimated area of total collagen (12.05 mm^2^) for epithelioid MM.

**Conclusion:**

Differential patterns in matricellular phenotypes in MM could be used in translational studies to improve patient outcome. More importantly, our data raise the possibility that cancer cells can use the matrisome for disease expansion and could be effectively targeted by anti-collagen, anti-elastin, and/or anti-hyaluronic acid therapies.

## Introduction

Malignant mesothelioma (MM) is a rare malignancy characterized by its aggressive growth, local invasion, and strong etiologic relationship to asbestos exposure. MM arises from serosal mesothelial cells, mesodermal derivative cells that exhibit both epithelial and mesenchymal features (1). Different clinical trials have investigated the striking differences in behavior and response to therapy in MM patients, some often suggest that this heterogeneity may emerge from the presence of different cell populations in a single tumor (2). The heterogeneous and aggressive nature of this tumor often lead to a poor prognosis for these patients. Therefore, it is crucial that we improve our comprehension of MM heterogeneity in its several aspects to develop new therapeutic protocols that can improve survival.

Tumor heterogeneity can be investigated at the intra-tumor and inter-tumor levels and contemplates not only tumor cell populations but also their microenvironments. There are currently three major histological MM types: epithelioid, biphasic (epithelioid-sarcomatoid), and sarcomatoid. The nonmalignant cells in the tumor microenvironment are, in turn, called the stroma and include blood vessels, immune cells, fibroblasts, signaling molecules, and the extracellular matrix (ECM) (3). Regarding the latter, it is worth noting that the ECM core matrisome can be broken down into fibrillar collagen types (such as types I), proteoglycans (such as hyaluronic acid), and glycoproteins (such as elastin) (4).

Previous histological observations made by pathologists identified two juxtaposed tumor cell populations (epithelioid and sarcomatoid) in MM. To further explore this question, our group adopted a dissecting approach to decompose each bulk MM histochemistry profile into a combination of these two cell populations. This novel approach quantifies different cell populations in a single tumor and avoids a strict subtype signature based on subjective hierarchical classifications that fail to take into account intermediate phenotypes and show intrinsic limitations when investigating intra-tumor heterogeneity. We also used bioinformatics to decompose and reconstruct MM profiles. This new method to classify the pathology is a step forward toward an improved comprehension of the underlying behavior of MM when different cell populations coexist in the same tumor. Moreover, this technique can have critical clinical applications and implications for prognosis and therapeutic strategies.

## Patients and Methods

### Patient's Selection and Clinicopathologic Review

This retrospective study was conducted using data from institutions linked to the Hospital das Clínicas Complex of the Faculty of Medicine of the University of São Paulo (HC-FMUSP)–including the Central Institute (Division of Pathological Anatomy, DAP), the Heart Institute (InCOR, Laboratory of Pathological Anatomy) and the Cancer Institute of the State of São Paulo (ICESP)–and was approved by their Ethics Committees (protocol number: 2,394,571).

A search for the word “mesothelioma” led our group to 246 cases treated between 2008 and 2018 in the three institutions−75 at InCOR, 59 at HC-FMUSP, and 112 at ICESP. However, not all cases identified in the search were MM cases, since the word “mesothelioma” was sometimes used in the comments section of differentiated diagnoses, as well as in reports related to benign mesotheliomas, their variants (papillary, well-differentiated cystic), and cytological exams. Other cases were dismissed due to their blocks not being found or having been removed for external review or because they did not meet the proposed inclusion criteria. The final sample totaled 87 cases (35.4% of the initial search), as shown in Supplementary Figure 1.

All blocks and slides of the cases initially found (246) were requested from the pathology files for review by two experienced pathologists in Thoracic and Pulmonary Pathology (VLC and MLB). The review compared the diagnosis and findings reported in the anatomopathological reports, including the immunohistochemical test, with the original slides. When the slides were lightened or showed preservation artifacts due to temporal wear, they were considered unsuitable for reassessment, and new cuts or immunohistochemical reactions were performed.

Moreover, these reviews used the diagnostic criteria reported in the World Health Organization (WHO) update of 2021 (5). MM cases were histologically classified by their predominant tumor cell population – epithelioid or sarcomatoid. The nuclear features, as described by Kadota et al. (6), are illustrated in Supplementary Figure 2. All cases had their immunohistochemical profile reviewed–and expanded when necessary–to ensure a minimum of two positive and two negative markers for MM, as proposed by the WHO (7, 8). Positivity was expected to fall between 80 and 100% for D2-40 and between 70 and 93% for WT1, whereas negativity was expected to fall between 95 and 100% for both D2-40 and MOC31. In case of any remaining uncertainty, the pathologists expanded the panel, evaluated it with the BAP1, and individualized each characterization according to the clinical context on a case-by-case basis.

We extracted clinical data from the original anatomopathological reports, imaging examination reports, surgical reports, and patient charts. Asbestos exposure was inferred from indirect data such as residential location and registered employment history. We also used search engines to search for each patient's name online, looking for indexing, litigations, or any association with groups of former workers in the asbestos industries. Next, we staged pleural mesotheliomas according to the clinical-pathological model of the 8th edition of the AJCC/UICC (8), whereas extrapleural cases were staged according to the patient's medical record. Overall survival (OS) was defined as the time interval between the date of histopathological diagnosis and the outcome event (death or end of segment, if alive) and was obtained from death records at a registry office or at a death verification service. If no death records were identified, the time of the last follow-up was calculated based on the last consultation or laboratory tests in the computerized hospital system.

Table 1 summarizes the clinical-pathological and epidemiologic data of these patients.

**Table 1:** Clinical characteristics of patients with malignant mesothelioma.

**Characteristic**	**Number (%)**
Age (years)^*^
Median (range)	60 (35–92)
**Sex**, ***n*** **(%)**
Male	59 (67.8%)
Female	28 (32.2%)
**Asbestos exposure**, ***n*** **(%)**
No	43 (49.4%)
Yes	44 (50.6%)
**Stage**^†^ **III/IV**	87 (100%)
**Treatment**, ***n*** **(%)**
Surgery	62 (71.3%)
Chemotherapy	25 (28.7%)
Overall Survival, median^*^	21.6 months
Status^*^
Alive	35 (41.7%)
Died	52 (58.3%)

* *Some cases lacked follow-up information: Age [3]; Overall Survival [16]; Status [3]*.

† *Per International Association for the Study of Lung Cancer (IASLC) criteria (7)*.

### Morphological Sample Assessmeny

#### Construction of Tissue Microarray (TMA)

We chose the TMA investigation model based on the currently available literature, including studies of immune response (9–11). Before to construct the model, we carefully examined the hematoxylin and eosin (HE) stained slides to assure that areas epithelioid and sarcomatoid were present. Then, three cylinders of 1.0 mm in diameter containing the epithelioid areas and three cylinders containing sarcomatoid areas, were noted on the original corresponding HE slides and paraffin blocks (named as “donor” blocks) and then extracted and transported to receiver paraffin blocks using the precision mechanized equipment MTA1 (Manual Tissue Microarrayer, Beecher Instruments, USA). Each cylinder was positioned in the receiver block according to a previously prepared map, with a 0.3 mm spacing between samples (Supplementary Figure 3). Each case produced six cylinders distributed in duplicate in the receiver block, aiming to minimize a possible sampling bias resulting from physical losses and/or representativeness inherent to the TMA technology. Next, the TMA blocks were submitted to serial 3 μm-thick cuts in a manual microtome (Leica Instruments, Germany), each cut made in a single session to avoid losses with trimming. As a result, each block produced 70 sections that were then distributed on a marked slide embedded in paraffin and stored in a dark box at−20°C to preserve the antigenicity of the samples. The built TMAs are illustrated in Supplementary Figure 4.

#### Histochemistry

Each MM TMA had one of its sections stained using the Modified Russell-Movat's pentachrome stain adopted by the FMUSP biotechnics sector (12). Supplementary Table 1 lists the evaluable connective elements and their respective color tones. Supplementary Figure 5 illustrates the elements of Movat's pentachrome stain under evaluation. We also subjected the paraffin blocks with a representative surgical specimen to a Picrosirius histochemical staining and visualized it under polarized light under 90 degrees for indirect identification of type I fibers (coarser in appearance, in shades ranging from yellowish to reddish) and type III fibers (more delicate and greenish) (13, 14).

#### Scanning and Image Capture of Histology Slides

Histology slides for brightfield viewing (HE, Movat, Picrosirius) were scanned in a Pannoramic 250 scanner (3DHistech, Budapest, Hungary), under a 40x objective (Plan-Apochromat, 40x/NA0.95, Zeiss, Germany), with a resulting pixel density of 0.185 μm2. The resulting files, saved in mirax format, were stored on an external hard disk with a 2 TB capacity, with redundant copies on a secondary disk for data security. For visualization, we used the proprietary software Panoramic Viewer (3DHistech) and QuPath open platform, version 0.2.0-m4 (Centre for Cancer Research & Cell Biology, University of Edinburgh, Edinburgh, Scotland). For scientific documentation and acquisition of microscopy images under polarized light, we used Zeiss Axiocam 512 scientific camera (Zeiss, Germany) coupled to a Zeiss Axioscope A1 optical microscope with x40 and x63 N-Planochromatic objectives (Zeiss) under the Zen 3.0 (Zeiss) software to acquire brightfield and polarized light images.

#### Computerized Semi-assisted Quantification

We used the QuPath analysis visualization software in a semi-assisted manner. This platform had been previously validated in other studies (15), and we followed the protocol suggested by their authors (16). After the data was uploaded to the software, QuPath normalized the slide vectors, “dissecting” epithelioid and sarcomatoid areas, corrected them by automated sample detection, delineating them (Supplementary Figure 6), and computer them as cellularity (Supplementary Figures 7, 8). We then quantified the Movat's stain in epithelioid and sarcomatoid areas associating the Trainable Weka Segmentation (TWS) machine learning tool (“Waikato Environment for Knowledge Segmentation”) (17) and the ImageJ software (National Institute of Health, USA). Next, a training set was created by an experienced pathologist (MLB) consisting of 18 images of 100 x 100 pixels extracted from the general sample. These images are representative of “ideal” areas (ground truth), representing the components highlighted in this coloration: fibrin, collagen matrix, hyaluronic acid, elastic fibers (Supplementary Figure 9). The correspondence of each of these elements was “taught” to the system through slide annotations, algorithms, trial and error, correction to its adequacy, and validation. Once the training set had been validated, the algorithm grouped all spots into separate images. This group segmentation resulted in 8-bit colored images censored by the previously designated color codes. Next, these images were again validated by a pathologist (MLB) and finally quantified by component under the optical threshold in the ImageJ software. The final measurements of cellularity, hyaluronic acid, fibrin, elastin, and total collagen in epithelioid and sarcomatoid populations obtained from the three cylinders in the TMA were averaged and directly calculated on the QuPath software. A final single patient value was expressed as the percentage per mm^2^, and then transferred to individual patients to determine OS and risk of death as final endpoint. Moreover, heterogeneity among the different cylinders from a same patient occurred mimicking the scenario of MM, a heterogeneous tumor. Albeit this heterogeneity, the predominant histoarchitecture was considered.

#### Three-Dimensional Reconstruction and 3D Collagen Printing

Type I and III collagen fibers were reconstructed using the Picrosirius histological staining, a technique based on the azo pigment Sirius Red F 3B in saturated picric acid, as described by Junqueira et al. (18). The purpose of this reconstruction method was to create a 3D visualization of the patterns found in collagen networks made up of Col fibers type I and III. We chose not to individualize them to better understand the spatial distribution between the neoplastic cell blocks. Other collagen types that could not be stained with Picrosirius were not reconstructed because the method was chosen for its affordability. In this coloration, when viewed under polarized light with a brightfield optical microscope, Col I fibers are identified as thick, reddish, or orange-colored fibers, whereas Col III fibers are thin and greenish. Supplementary Figure 10 is a photomicrograph that highlights the observed patterns of staining with or without polarized microscopy. In the absence of polarization, all collagen fibers had a reddish color, contrasting with the yellowish tones of the cytoplasm and muscle tissue. It was only under the use of polarized light that, as previously mentioned, the different fiber refringence patterns, conformations, and color patterns between Col I and III emerged. Also, lower magnification showed their architectural distribution as ECM components, with different patterns of fiber distribution: Col (I) was organized in thicker orange and reddish fibers, whereas Col (III) fibers were thin and greenish.

The collagen reconstruction involved the use of a destructive microscopy technique (19) where ten 3μm sequential cuts are made in the paraffin blocks of surgical specimens containing viable tumor cell representation and ECM. All the block slices are then stained using the Picrosirius red technique in a single session to avoid technique variations. Next, their images are captured using a brightfield optical microscope (Zeiss Axioscope A1, Zeiss), with 4x (N-Achroplan NA: 0.15, Zeiss), 20x (N-Achroplan, NA: 0.45, Zeiss) and 63x (Achroplan, NA: 0.56, Zeiss) magnification, polarizer, and led light source. The camera employed in this study was a scientific camera with a 12-megapixel, 1-inch CCD sensor, Axiocam 512 (Zeiss). The images were captured in multiple magnitudes, sequentially, at the same point on all slides, and the image files were saved in the proprietary format of the Zen 3.0 capture software (Zeiss) “.czi” and exported in uncompressed “.tif” format, with 100% quality. For the collagen reconstruction, we used the images captured under polarization and under 4 and 20x objectives. The next step was to align the images digitally. First, an image grouping (stack) was imported into the Fiji software and transposed to 8-bit in grayscale. We then applied the optical threshold (threshold) of the Otsui method to highlight Col I and III fibers and aligned them using the TrakEM2 (20) plugin, choosing the stack alignment option, without deformations, in the proposed configurations, with the affine transformation method. After a visual validation of the alignment, the resulting images were exported in “.tif” format. For the three-dimensional visualization, we used the Fiji software's 3D viewer and the 3D Slicer software (version 4.10.2 r28257) (21). We first imported the previously treated “.tif” images into the 3D Slicer software and defined a virtual spacing of 5 mm in the voxel metadata configuration for z-axis visualization. Then we established a similar optical threshold to the one used for collagen fibers through the threshold option, defined the plane filling, and carried out the smoothing treatment. Finally, the Fiji software viewer created the final 3D visualization and exported it to “.stf” format in 1.9 gigabytes files. Supplementary Figure 11 illustrates the image resulting from the reconstructions by Fiji (A) and 3D Slicer (B) software. With this file, the next step was to prepare it for 3D printing, reducing the image's vertices and triangles. Since the original image had 30966169 vertices and 61915216 triangles, stored in 2.88 gigabytes, the resolution of the triangles was reduced to achieve printability, without loss of quality in the perception of the reconstruction. To do so, we used Autodesk Meshmixer (Autodesk, USA) (22, 23) to create a “.stl” file of 18.8 megabytes containing 116,147 vertices and 234,066 triangles, with dimensions of 100.00 x 65,713 x 22.203 mm. In addition, the model was simplified by excluding loose stitches, that is, those without connection to other stitches, and rounding of the ends for printing, as illustrated in Supplementary Figure 12. Once adjusted for printing, the model was submitted to the Craftcloud website^1^ for printing on a resin printer with a resolution of 0.05 mm.

### Data Analysis

The statistical analysis was performed using SPSS v18 (Chicago, IL, USA) for Windows. We assessed the relationship between quantitative variables using Student's *t*-test and used an analysis of variance to correlate the color patterns. The paired-sample *t*-test and general linear model were used to test the relationship between one continuous variable and several others. All patients were clustered for similar expression levels between the five morphometric variables (tumor cellularity, hyaluronic acid, fibrin, elastic fibers, and total collagen) on an R statistical software using the pvclust package which provides a bootstrap agglomerative hierarchical clustering option. Clusters with similar expressions of the five variables were analyzed for risk of death and survival time. The risk of death was obtained by logistic regression. The total accumulated survival time was calculated by the Kaplan-Meier method and analyzed by the log-rank test. A *P*-value of two seams <0.05 was considered statistically significant for all tests.

## Results

Table 1 summarizes the clinical characteristics of patients who were mostly male (67.8%) at a median age of 60 years. All patients were stage III/IV, 71.3% had undergone surgical resection, and 28.7% had received chemotherapy. 50.6% of patients reported prior exposure to asbestos. 58.3% of patients died after disease progression.

**Figure 1:**
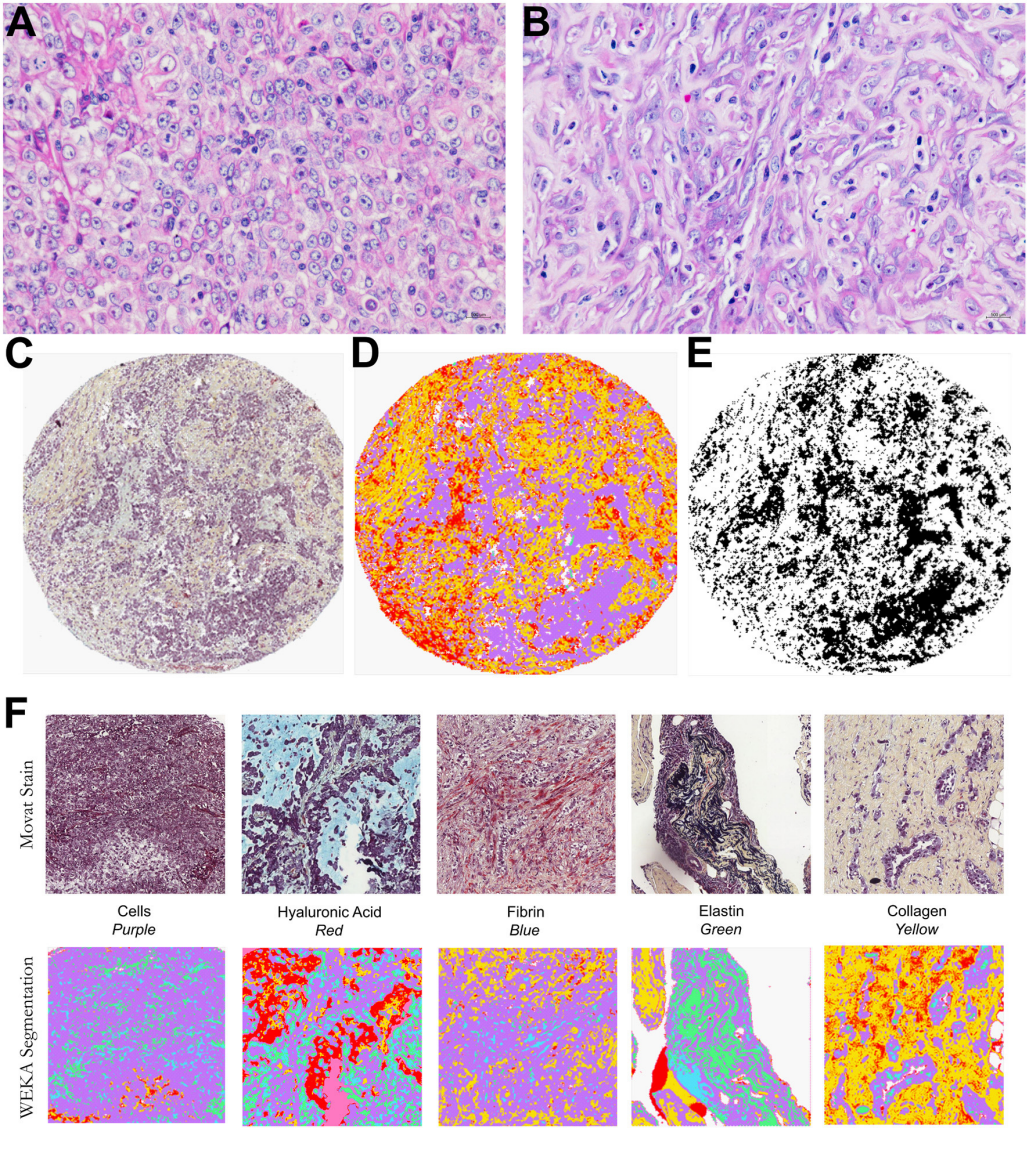
Histological characterization of a malignant mesothelioma (MM) cohort. Epithelioid **(A)** and sarcomatoid **(B)** MM stained by HE. Input images **(C)**, TMA spot stained by modified Russell-Movat staining; **(D,E)** output of the Weka segmentation and of the threshold segmentation for data extraction by coloration. **(F)** Input and output images of the Weka segmentation, showing areas of segmented fundamental truths.

Histological examinations found two contrasting tumor cell populations (epithelioid and sarcomatoid) in the MM cohort stained with Movat's pentachrome for cellularity and overall matrix characterization (Figure 1). The epithelioid population showed a prominent tumor cellularity involvement in the dense hyaluronic acid matrix. In contrast, the sarcomatoid tumor cell population had modest tumor cellularity and hyaluronic acid area fraction.

**Figure 2:**
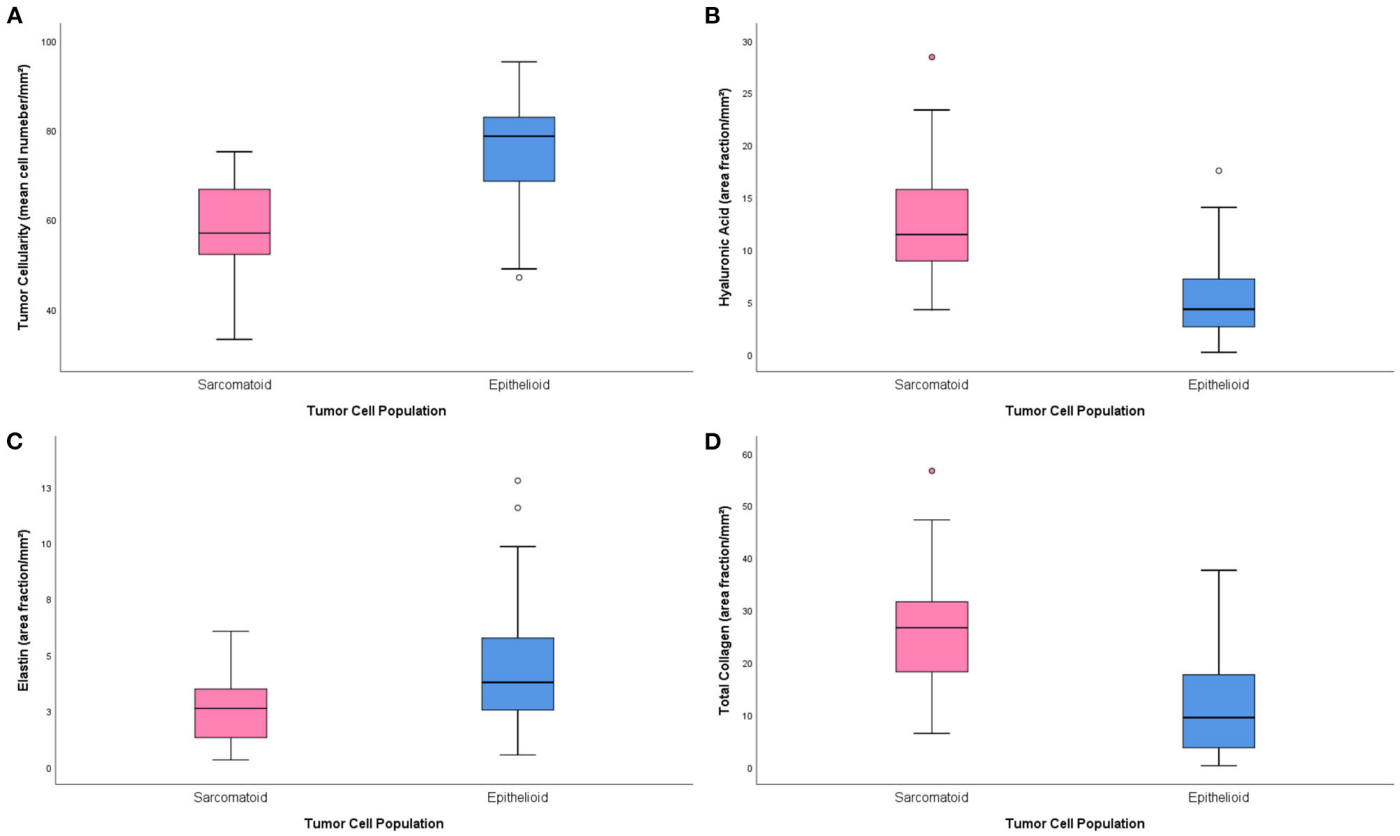
Boxplots of the distribution of matrix elements [cellularity **(A)**, hyaluronic acid **(B)**, fibrin **(C)** and total collagen **(D)**] between the epithelioid and sarcomatoid groups.

Table 2 brings the distribution of the matrix components in the epithelioid and sarcomatoid tumor cell populations of MM. A closer evaluation of the ECM through the elements of Movat's pentachrome stain showed two distinct profiles: the epithelioid cell population had a higher mean cell density (1.33 times higher than sarcomatoid), with higher matrix composition of elastic fibers (2.64 times higher), and fibrin (3.2 times higher). Conversely, hyaluronic acid, a non-fibrillary element of ECM, and total collagen were predominant in the sarcomatoid tumor cell population (1.63 and 2.71 times higher, respectively). Figure 2 uses four plots to compare the expression of matrix elements, including cellularity (Figure 2A), hyaluronic acid (Figure 2B), fibrin (Figure 2C), and total collagen (Figure 2D), between epithelioid and sarcomatoid tumor populations. The box plots in Figure 2A demonstrate a relatively strong relationship between cellularity and epithelioid tumor cell population (*P* = 0.0001), whereas the boxes in Figures 2B,D show a strong relationship between the sarcomatoid tumor population and hyaluronic acid and total collagen (*P* = 0.05 and *P* = 0.0001, respectively).

**Table 2:** Differences in the decomposed extracellular matrix factors between the epithelioid and sarcomatoid cell populations in MM.

	**Epithelioid Cell Population**	**Sarcomatoid Cell Population**	*P*-value^*^
Cellularity (mean cell number/mm^2^)	71.14	53.22	0.0001
Hyaluronic acid (area fraction/mm^2^)	6.57	10.73	0.05
Fibrin (area fraction/mm^2^)	4.13	1.29	0.0001
Elastin (area fraction/mm^2^)	6.08	2.30	0.0001
Total collagen (area fraction/mm^2^)	12.05	32.71	0.0001

* *The t-test was used to detect differences in continuous variables between groups of the tumor cell population. P-value ≤ 0.05 was considered statistically significant*.

**Figure 3:**
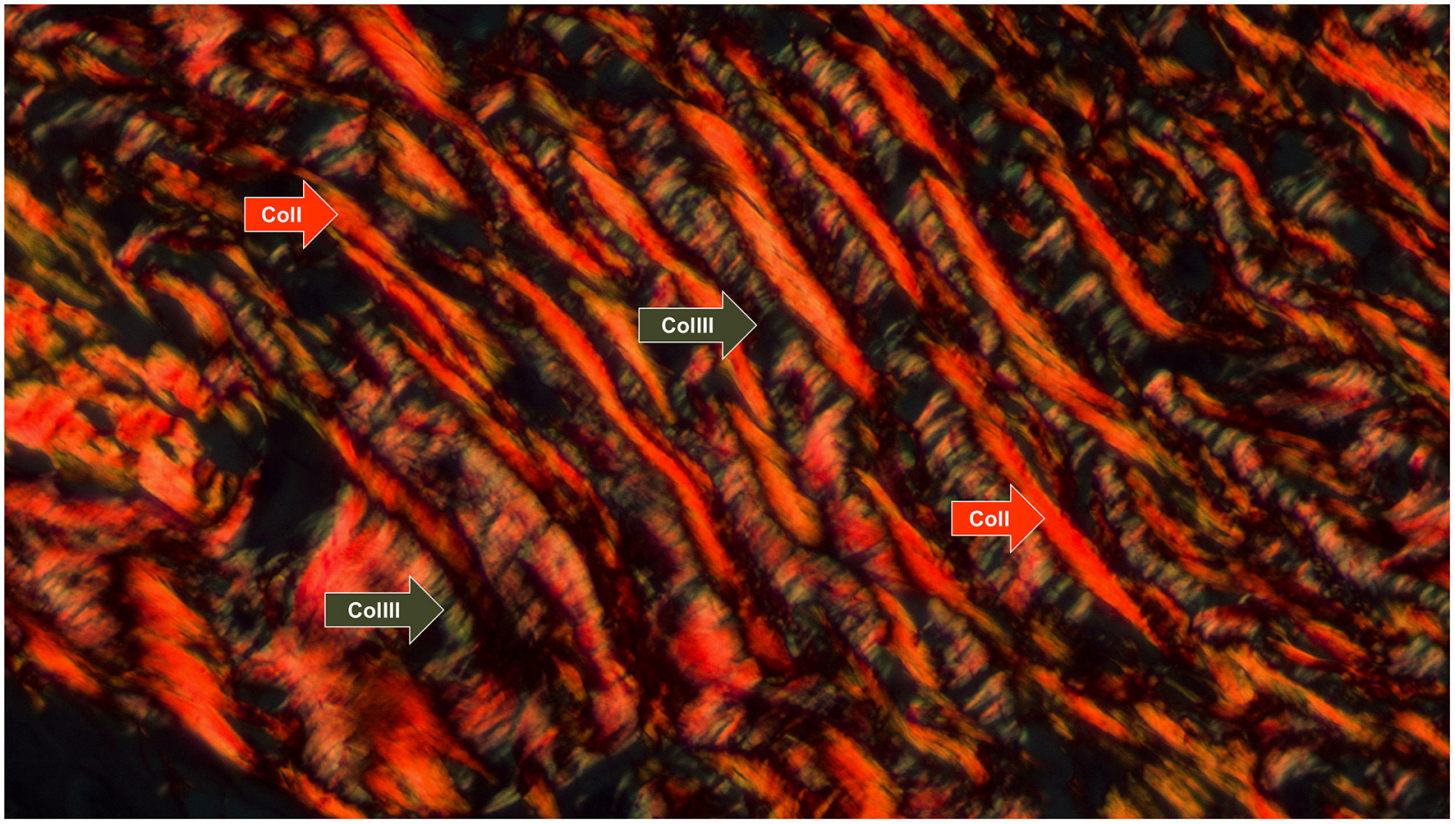
Collagen fiber patterns I and III stained with picrosirius red and observed under polarized light. Type I fibers (thick and reddish fibers) and type III (thin and greenish fibers) are indicated by the arrows (red and green, respectively) (Picrosirius under polarized light, 630x).

**Figure 4:**
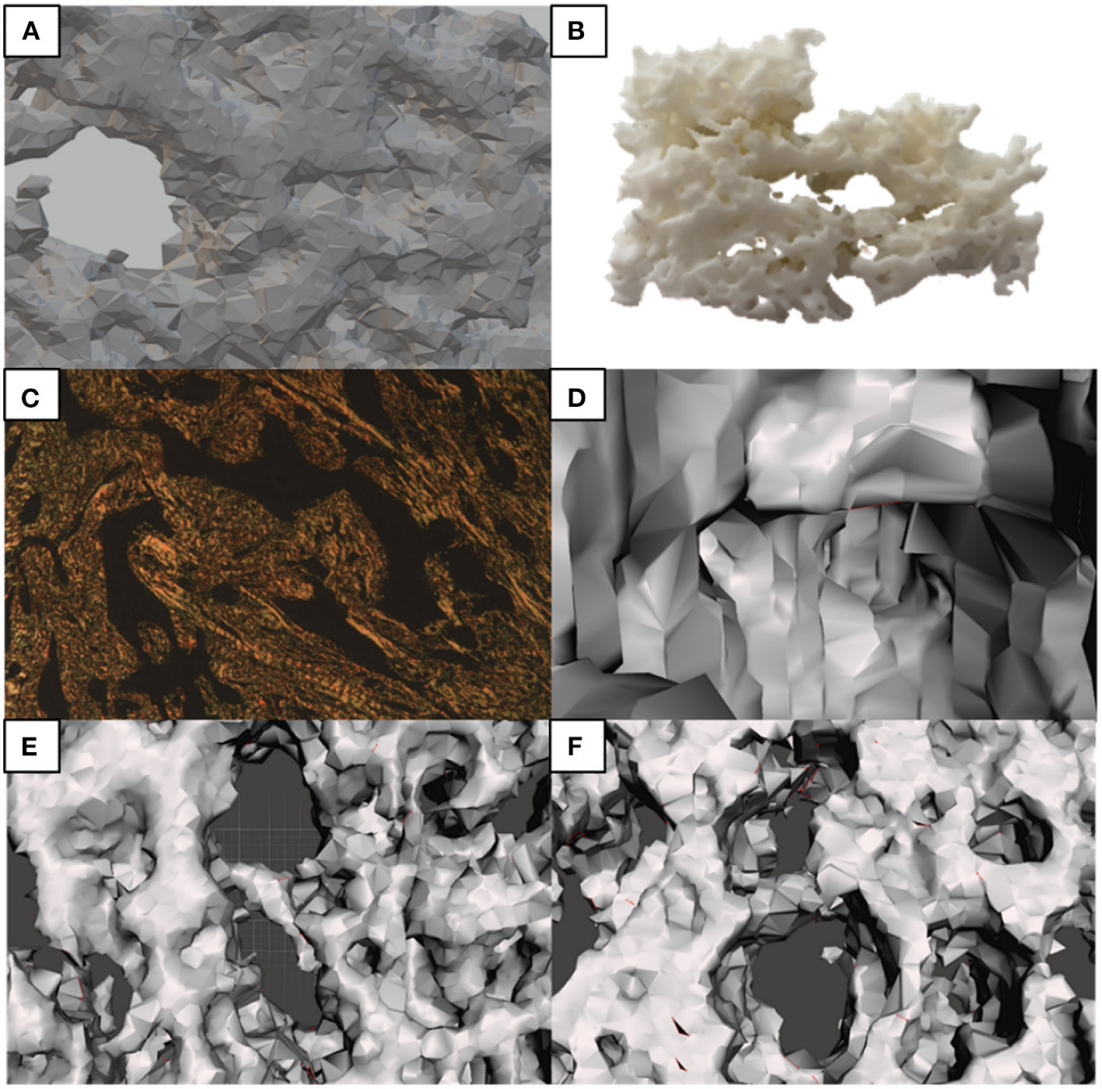
Morphological characteristics observed under conventional microscopy and identified in the 3D model. **(A)** Simplified 3D model; **(B)** 3D printed model; **(C)** 2D microphotography–Picrosirius; **(D)** Features in the 3D model, such as the communicating channels pointed; **(E,F)** are the equivalent of the thick central septa of **(C)**, seen from different angles in the 3D model.

The three-dimensional reconstruction of the ECM based on Picrosirius made Col I and III more visible; the estimated volume of the two collagen fractions was 14% of the total volume in the chosen block, consistent with the median estimated volume of total Col (12.05) for epithelioid tumor population (Figure 3). The digital model was simplified to allow for three-dimensional printing and remove disjointed structures. As a result, Figure 4 shows features that were not observed by the two-dimensional brightfield optical microscopy, such as channels formed by Col fibers surrounding the neoplastic cell blocks. While the digital model showed cellular channel areas between collagen fibers, the printed model made ECM more tangible, as illustrated in Figure 4B.

**Figure 5:**
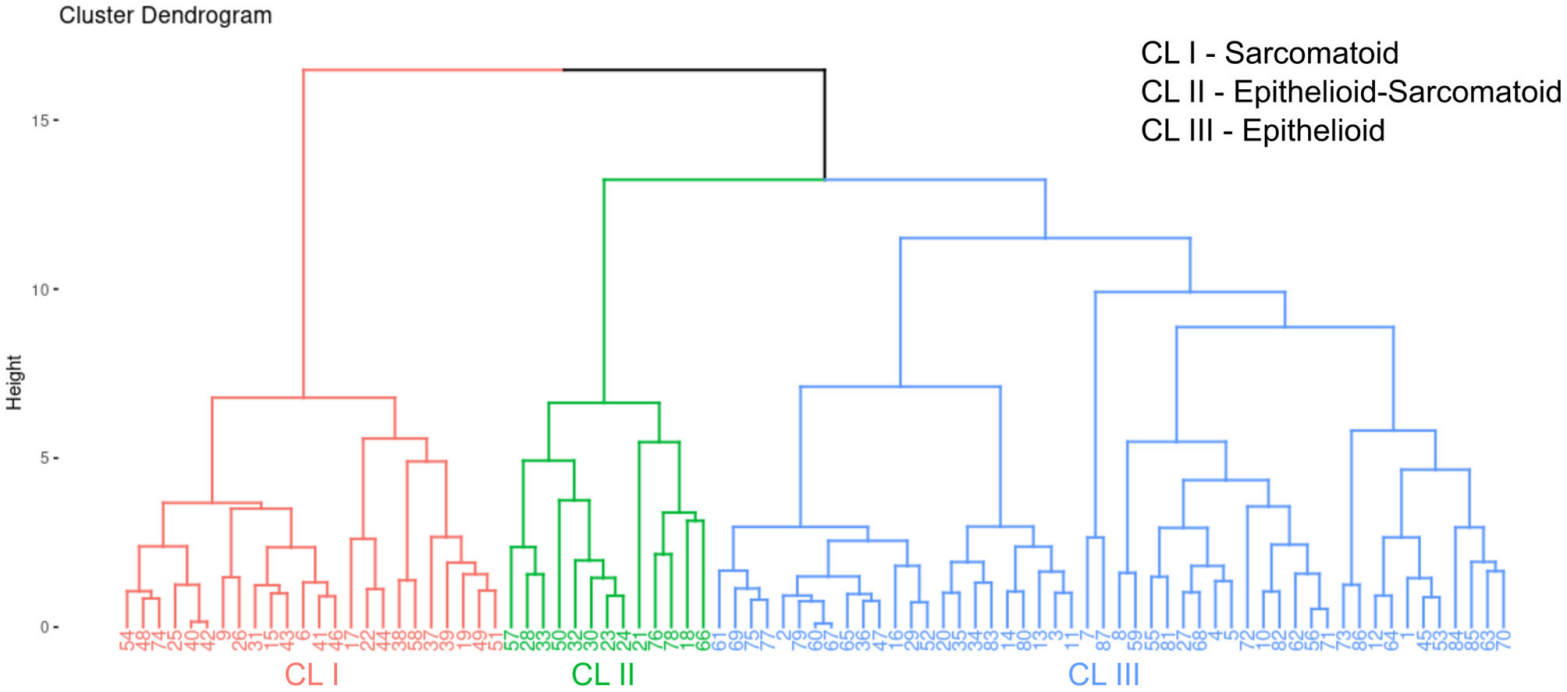
A dendrogram of clusters with three distinct groups split by dispersion similarities in our MM cohort.

**Figure 6:**
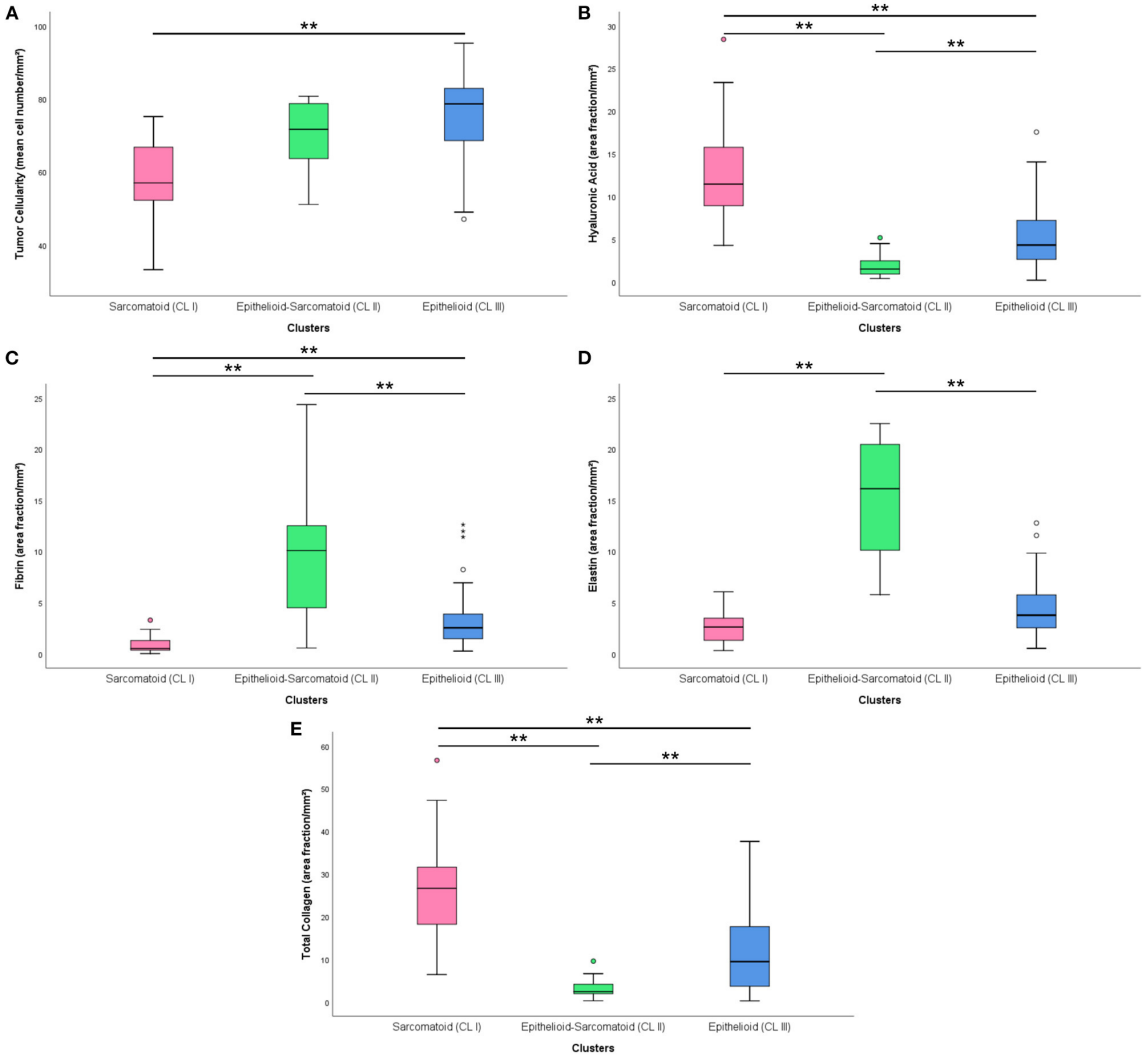
Boxplots with the distribution of matrix elements [cellularity **(A)**, hyaluronic acid **(B)**, fibrin **(C)**, elastin **(D)** and total collagen **(E)**] in different clusters. (**) was used when *P* ≥ 0.01.

After the univariate analysis showed which morphometric variables differed significantly between epithelioid and sarcomatoid tumor cell population in MM (tumor cellularity, hyaluronic acid, fibrin, elastic fibers, and total collagen), we grouped these variables in hierarchical cluster analyses independent of clinicopathological variables and identified three clusters of patients: 24 subjects in cluster 1 (CL I), 13 in cluster 2 (CL II), and 50 in cluster 3 (CL III). Figure 5 shows the cluster dendrogram separating the three groups by dispersion similarities. CL I included tumors with a high area fraction of hyaluronic acid (13.03/mm2) and total collagen (25.48/mm2) compared to CL II (1.97 and 3.30/mm2, respectively) and CL III (5.43 and 11.90/mm2, respectively) (Figure 6); this cluster coincided with sarcomatoid tumor cell population histology. In contrast, CL II had tumors with a high area fraction of fibrin (9.82/mm2), and elastin (15.37/mm2) than CL I (0.97 and 2.63/mm2, respectively), and CL III (3.36 and 4.41/mm2, respectively); suggesting a biphasic tumor cell population in CL II–that is, one that includes both epithelioid and sarcomatoid cell types (Figure 6). Finally, CL III was made of tumors with a high number of cells/mm2 (74.89 cells/mm2) compared to CL I (57.89/mm^2^) and CL II (69.54/mm^2^) and coincided with the epithelioid tumor cell population histology (Figure 6).

In Supplementary Table 2, Supplementary Figure 13 are shown the association between the three clusters classification with the final histotype (epithelioid, biphasic or sarcomatoid) resulting from pathological classification. Interestingly, cluster analysis recognizes with strong significance three different subsets in epithelioid MM classified only by histology (*X*^2^*; P* = 0.02).

Considering that BAP1 is a surrogate marker for the presence of BAP1 gene alterations, clusters classification was compared with BAP1 protein status. The distribution of BAP1 protein was positive in 17 (22%) epithelioid-sarcomatoid and 3 (3.9%) epithelioid histotypes (Supplementary Table 3, Supplementary Figure 14; *X*^2^*; P* = 0.48).

Table 3 shows the independent association between these clusters and survival probability in MM. CL II had three times the probability for better overall response (OR = 3.462, 95% CI = 1.115–10.746, *P* = 0.032).

**Table 3:** Unconditional logistic regression exploring the independent association of clusters categorization and survival risk.

	**B**	**S.E**.	**Sig**.	**Exp(β)**	**95% C.I. for Exp(β)**	
					**Inferior**	**Superior**
Cluster 3 (Reference)			0.064			
Cluster 1	0.143	0.684	0.834	1.154	0.302	4.406
Cluster 2	1.242	0.578	0.032	3.462	1.115	10.746
Constant	−1.099	0.436	0.012	0.333		

*B, coefficient for survival; S.E, standard error; Sig., significance; Exp (β), risk of B coefficient; C.I., confidence interval*.

**Figure 7:**
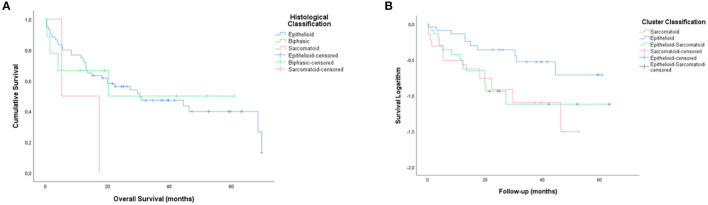
Kaplan-Meier curves showing different average survival times between the three clusters groups with those resulting from the histopathological classification of the cases into the three major histotypes (**B,A**, Log rank, 5.1 vs. 2.52; *P* < 0.01 vs. *P* = 0.28).

Figure 7 shows overall survival data compared cluster classification with those resulting from the histopathological classification of the cases into the three major histotypes. The median overall survival between the cases classification was respectively 30.1 vs. 37.6 for CIII and epithelioid histotype, 44.4 vs. 34.4 for CII and epithelioid-sarcomatoid histotype and 23.3 into three major histotypes was 11.4 months for CL I, 5.5 months for CL II, and 25.1 months for CL III. And 30.1 vs. 11.26 for CI and sarcomatoid histotype. Clearly, the clusters tended to separate patients into three groups with distinctly different average survival times compared to histological classification, as illustrated by Kaplan-Meier curves in Figures 7A,B. CL III appears as the top curve. By contrast, those in CL II and I overlapped (bottom curves), respectively (*P* < 0.01; by Log Rank test).

## Discussion

Our study described histologic studies of MM, the different tumor cell populations in these samples, and their extracellular matrix components. We then suggested a complementary way to describe MM behavior using Movat's pentachrome stain and the TWS bioinformatics approach [IP1].

Movat's pentachrome was described in 1955 as a histochemical technique to highlight multiple components of the connective tissue compartment (24); in 1972, it was modified by Russell (12), who optimized the technique. The pentachrome adds elements with different colors, such as Verhoeff, sodium thiosulfate, acetic acid, alcian blue, Scarlet orcein with acid fuchsin, and safro-alcohol solutions. This histochemistry staining technique produces massive image datasets when observed under an optical microscope. However, to quantitatively evaluate the images, researchers frequently need to manually annotate the components of interest, a time-consuming procedure. To overcome this problem, the TWS works as a machine learning tool that studies a restricted number of manual observations and creates a list of classifier elements to slice the remaining data automatically (17). The TWS approach breaks down different MM profiles–each made of a distinct combination of tumor features–and reconstructs them according to the different cell populations found in the samples, as well as their non-tumoral extracellular matrix. This approach also minimizes the number of requirements assessed in various MM histological subtypes and is driven by the occurrence of epithelioid and sarcomatoid morphologies in different proportions within MM. TWS can also cluster the samples through unsupervised segmentation learning schemes and can be tailored to employ user-designed image features or classifiers. Both Movat's pentachrome stain and the TWS depend on the premise that distinct morphological phenotypes correspond to distinct molecular phenotypes. Therefore, we infer that the dissecting approach may influence the potential improvement of clinical management in terms of prognosis or therapeutic plan.

Along these lines, our results highlight several crucial points, namely: the combination of different tumor cell components, their relationship with their microenvironment, their association with patient survival, and their possible contribution to defining new therapeutic strategies. Using a similar approach, Mäkelä et al. (25) reported the prognostic value of fibroblast foci and inflammation in idiopathic pulmonary fibrosis. In an elegant study, Jones and colleagues applied an integrated micro-CT and Movat's stain to dissect the morphology of fibroblast foci in 3D and reveal a collection of heterogeneous structures, suggesting previously unrecognized plasticity, in contrast to the 2D tissue standard pathology of sections (26). Blum et al. (27) employed WISP, a deconvolution method, to show that different morphological phenotypes in MM correspond to distinct transcriptome phenotypes. More recently, Jargidar et al. (28) tested epithelioid, biphasic and sarcomatoid MM cell types *in vitro* and found that fibronectin (FN) and homologous cell-derived extracellular matrix (hcd-ECM) treated substratum differentially affected the above phenotypes; 3D MM spheroid invasion was higher in fibronectin-collagen matrices in the epithelioid and biphasic cells, while 3D cell cultures of epithelioid and sarcomatoid MM cells in fibronectin-collagen showed a higher contractility compared to hcd-ECM-collagen. Collectively, these results support our findings that histological subtypes are remarkably consistent with the MM-derived epithelioid-like, biphasic-like, and sarcomatoid-like tumor cell populations. Using thresholds to discriminate these populations, we can equally recapitulate all former tumor classification systems. We suggest that our method offers a more objective solution for describing MM subtypes, in contrast with discrete classification systems based either on morphology or molecular parameters to modulate stratified clinical trials. We also believe that precision medicine may benefit from the finely tuned information provided here to establish, for instance, drug combinations and dosages that target different tumor cell compartments.

To understand the relationship between different tumor cell populations and their matricellular factors in MM progression, we used a three-stage design. First, we used histochemistry and a computerized semi-assisted quantification system to characterize the ECM matrisome (fibrin, hyaluronic acid, elastin, and collagen) in both epithelioid and sarcomatoid tumor cell populations. Second, the components that showed significant differences between epithelioid and sarcomatoid populations, regardless of any clinicopathological variables, were grouped according to the similarities produced in high-throughput protein analyses and used to characterize different subgroups of MM. Third, we examined the clinical association between different tumor-matricellular factors in TMAs built from 87 patients with surgically excised MM. Finally, we showed that this more subtle way of characterizing different tumor cell populations and stroma context provides a better understanding of the clinical behavior of MM.

However, some major points still require further investigations. The first important question that remains unanswered pertains to the significance of the high area fraction of fibrin and elastosis found in the ECM of the epithelioid cell population when compared to the sarcomatoid population. The behavior of individual cells is dictated by the forces exerted on them by the surrounding ECM (29), and the physical attachments that connect the cell interior to the ECM (30). During oncogenesis, the tumor stroma is changed, suffering modifications in its biochemical and viscoelastic properties, including elastic fibers (31). The ECM in solid tumors is stiffened, and as the tumor mass grows, it induces tumor hypoxia and cellular injury due to the increased interstitial pressure (32) modulating tumor cell phenotype (33). It has also been established that the stiffened ECM is not an inactive bioproduct of cellular dedifferentiation but rather a dynamic contributor of tumor growth and progression (33). Augmented ECM stiffness and increasing core matrisome factors also disrupt stroma morphogenesis, thus helping develop a specific malignant phenotype in tumor cell populations (34). To understand the dynamic nature of the ECM and the functional consequences of ECM changes as tumor tissues develop and remodel, we can use the classic concept of tissue regeneration, repair, and remodeling (3Rs) as an example (35). In the repair stage, edema, cytokines, and growth factors originating from the opening of endothelial cells' tight junctions lead to a “fibrillar” network composed of plasma proteins, such as fibrinogen and fibrin (36). These plasma proteins intermingle to form a crossed-linked gel which works as a temporary scaffold for cellular regeneration after injury (37). Thereby, the provisional matrix confers a framework and substrate for other cells, such as fibroblasts, which characterizes the remodeling stage. In MM, this substrate modulates the typical phenotype of the epithelioid tumor cell population in contact with this matrix.

Among the components studied, elastin helps define the rigidity and elasticity of the regular ECM, while fibrin forms the scaffold to support neoplastic cells (38). Elastin, one of the longest-lived proteins (39), is highly present in tissues subjected to high mechanical stresses and demands recurrent cycles of extension and contraction such as the skin, lungs, tendons, or arteries (40). During oncogenesis, tropoelastin degradation leads to the release of elastokines, bioactive elastin-derived peptides. Elastokines modulate tumor cell phenotype by exciting several properties of tumor cells, including a higher expression and secretion of proteases that powerfully stimulate tumor cell migration and matrix invasion (41). It has been reported that elastokines have intense chemotactic activity on malignant melanoma cells, and their presence in distant organs might contribute to metastasis (42). Elastokines have also been shown to promote *in vitro* proliferation of glioblastoma cells (43) and astrocytoma human cell lines (44), as well as murine melanoma cell lines (45). Using the Elastin van Gieson (EVG) stain, Al Abri et al. (46) reported that the amount of elastosis varied in different breast cancer cell populations and could be considered a surrogate marker for estrogen positivity and HER2/neu negativity in breast cancer patients. Using a similar approach, Kardam et al. (47) quantitatively evaluated elastic fibers stained by Verhoeff–Van Gieson in oral squamous cell carcinoma and found different grades of elastosis involved in disease progression.

The second question that should be further investigated involves the hyaluronic acid (HA) and total collagen densely present in the ECM of sarcomatoid tumor cell populations, contrasting with only a minor area fraction in the stroma of epithelioid populations. In short, HA is a ubiquitous connective tissue glycosaminoglycan synthesized by HA synthase enzymes (48) that supports matrix stability and tissue hydration. HA is also known to self-associate to form fibers (cables), networks, and stacks (36). At the cell surface, HA forms a huge pericellular matrix or “coat” named “glycocalyx,” which plays several critical roles, from morphology and mechanochemical functions to cellular cycle regulation and motility (49). This cell coat allows the cells to change shape and facilitates cell division and migration (50), which explains why it was more highly expressed in the sarcomatoid tumor cell population. It has also been reported that ECM cytoskeleton components have both tumor-suppressing and tumor-promoting capacities, and depending on its molecular weight, HA may work as either a tumor suppressor or a tumor promoter (49). As previously demonstrated by Tian et al. (51), naked mole-rat's tumor resistance involves the presence of a unique HA with high molecular mass as a major component in their ECM. This HA with high molecular mass signals through the CD44 receptor and triggers the expression of crucial tumor suppressor genes, promoting a hypersensitive cell-cycle arrest, a usual mechanism of tumor suppression (52). Conversely, high levels of small HA oligosaccharides are associated with poor prognosis in several tumors such as colorectal, breast, and prostate cancer (53–55). Collectively, these data contribute to understanding our findings of a greater amount of HA in MM sarcomatoid cell populations. HA interactions are prominent in diseases such as cancer and affect events that promote tumor cell phenotypes with higher invasion and metastasis rates (56). In fact, changes in cell shape are one of well-proved methods that cancer cells use to overcome mechanical barriers and thus competently invade restricted networks (57).

Interestingly, interactions between HA molecules and fibrillar collagen types seem to modulate the mechanical function of the collagen and modify the contractile forces produced by fusiform sarcomatoid cells (58). Moreover, the release of mechanical forces in collagen fibers, which seems to be dependent upon the synthesis and secretion of HA, have been linked to myofibroblast loss (59), suggesting that pericellular HA may thus promote myofibroblast survival and, consequently, collagen synthesis. In the current study, we observed inhomogeneities in 3D collagen matrices that reflected the mechanical phenotype of the matrices. We also observed an adjustment between pore size and stiffness, a critical factor for invasion (60, 61). Therefore, we successfully gained insights about structural cytoskeletons and mechanical properties of the tumor stroma, as these support the invasion of the sarcomatoid tumor cell population.

Finally, a third important question that should be addressed in future research relates to the need of a more accurate diagnosis of MM. MM has gained importance because of its association with exposure to asbestos, which has become more prevalent in recent years. However, the current fear of litigation in MM diagnoses has implications in prognosis and therapeutic protocols. The difficulties regarding MM are also intensified beyond the usual problem of anaplasia by its many subtypes and underlying substrate dimorphism (62). While knowledge of the more frequent histologic patterns has improved diagnosis precision, a great deal of inter observer subjectivity remains necessary (63). In this context, the use of a clustering method to collect more objective data is desirable. When we compared how the three clusters correlated with the final histotype (epithelioid, biphasic or sarcomatoid) resulting from pathological classification, we found that cluster analysis recognizes with strong significance three different subsets inside of epithelioid MM classified only by histology. We also found that the distribution of BAP1 protein was positive in 17 (22%) epithelioid-sarcomatoid, speculating a better behavior for those patients? Moreover, comparison between survival curves obtained with cluster and histopathological classification showed that histochemistry evaluation of matrix refined the prognostic information, suggesting that both procedures should be combined in the routine practice. Interestingly, our cluster analysis identified three groups of MM with prognostic implications: CL II (low risk of death), CL I (intermediate risk of death) and CL III (high risk of death). Overall, our results showed that the decreased risk of death in CL II patients was characterized by an epithelioid-sarcomatoid (biphasic) tumor cell population. In fact, these patients had a three times higher chance of survival than patients in CL I (epithelioid) and CL III (sarcomatoid). The reasons for this difference may be linked to a better balance between components that favor invasion (elastin, HA and collagen) and those that act as a barrier (cellularity).

In summary, these developing mechanisms help investigators to better characterize the phenotypes and functional mechanisms of tumors that express different cell populations. The characterization of distinct cell populations using specific biomarkers, microdissection, or single cell analysis is an incredibly exciting field with many questions that are yet to be answered. To contribute to these efforts, our study tests new methods of analyzing MM tumor cell populations and complements the description of MM behavior by integrating different tumor cells population and their extracellular matrix components. Notably, our findings may help guide more personalized treatments for MM patients and help develop novel targeted therapies, while also highlighting new ways for other researchers to investigate MM treatments.

## Data Availability Statement

The original contributions presented in the study are included in the article/Supplementary Materials, further inquiries can be directed to the corresponding author.

## Ethics Statement

The studies involving human participants were reviewed and approved by Ethics Committees of the Hospital das Clínicas Complex of the Faculty of Medicine of the University of São Paulo (HC-FMUSP), the Heart Institute (InCOR, Laboratory of Pathological Anatomy), and the Cancer Institute of the State of São Paulo (ICESP), approve by protocol number: 2,394,571. Written informed consent for participation was not required for this study in accordance with the national legislation and the institutional requirements.

## Author Contributions

VC and MB: conception and design. VC, MB, and CB: writing, review and editing. VC, MB, CB, AV, and WT: data analysis and interpretation. VC, MB, CB, and CF: statistical analysis. MB, AKA, and AMA: provision of study materials or patients. VC: administrative support. All authors: final approval of manuscript.

## Funding

This work was supported by São Paulo Research Foundation (FAPESP; 2018/20403-6), the National Council for Scientific and Technological Development (CNPq; 483005/2012-6), and Coordenação de Aperfeiçoamento de Pessoal de Nível Superior-Brasil (CAPES; Finance Code 001).

## Conflict of Interest

The authors declare that the research was conducted in the absence of any commercial or financial relationships that could be construed as a potential conflict of interest.

## Publisher's Note

All claims expressed in this article are solely those of the authors and do not necessarily represent those of their affiliated organizations, or those of the publisher, the editors and the reviewers. Any product that may be evaluated in this article, or claim that may be made by its manufacturer, is not guaranteed or endorsed by the publisher.

## Footnotes

^1^www.craftcloud.com, Germany
