# Supplementary figures and images for: Dissecting and Reconstructing Matrix in Malignant Mesothelioma Through Histocell-Histochemistry Gradients for Clinical Applications
Source: Front Med (Lausanne). 2022 Apr 13;9:871202. doi: 10.3389/fmed.2022.871202 (PMC9043486; doi:10.3389/fmed.2022.871202)

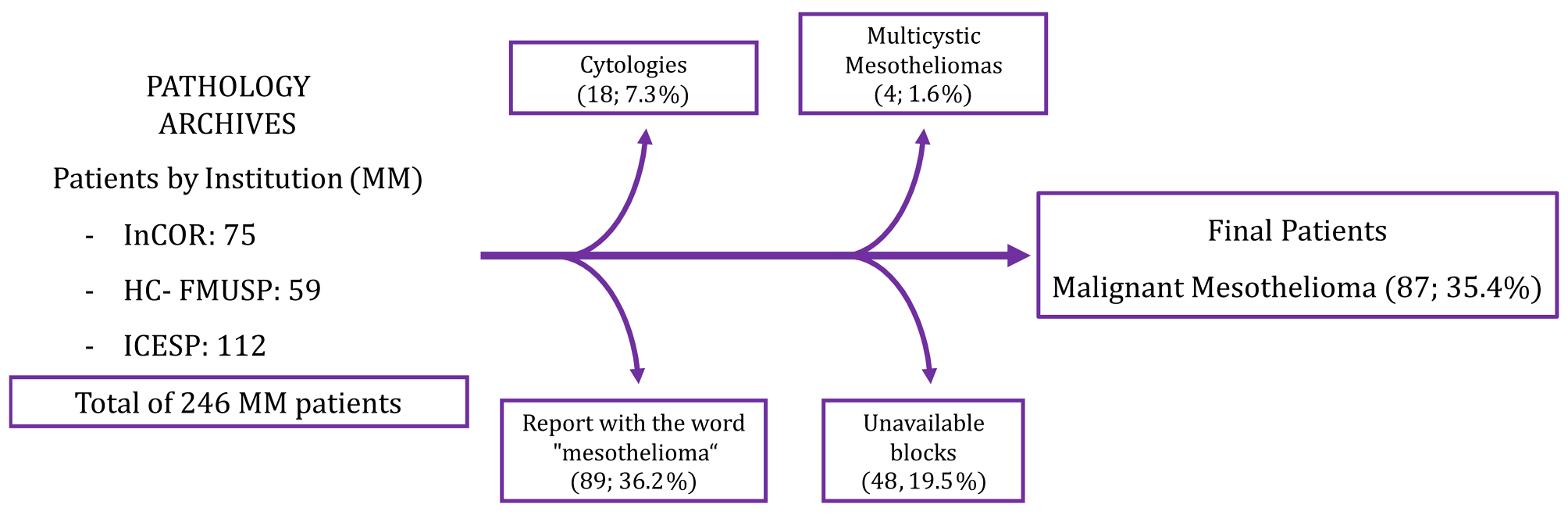

Supplement: Supplementary file 1 [file Image_1.TIFF]

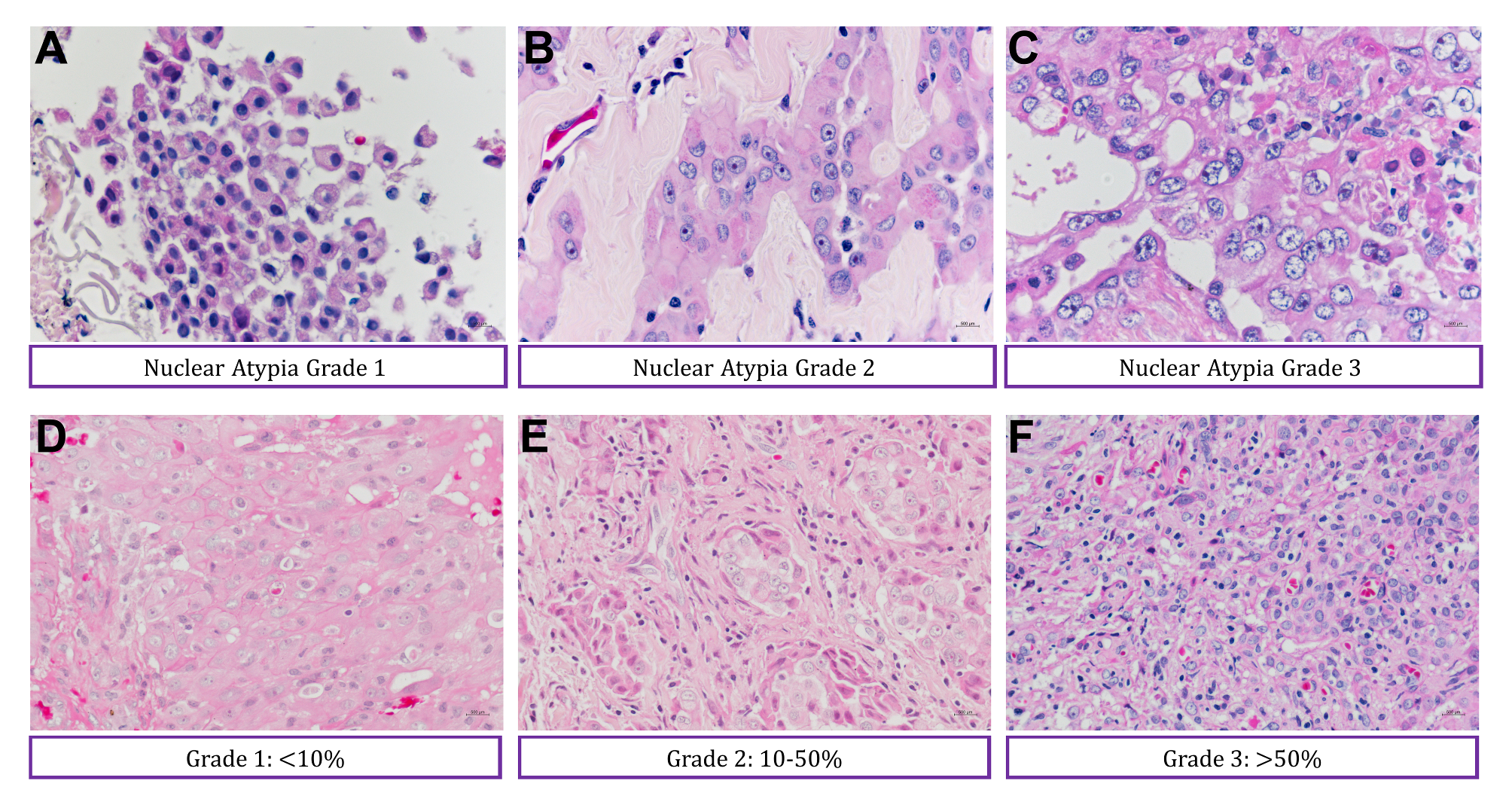

Supplement: Supplementary file 2 [file Image_2.TIFF]

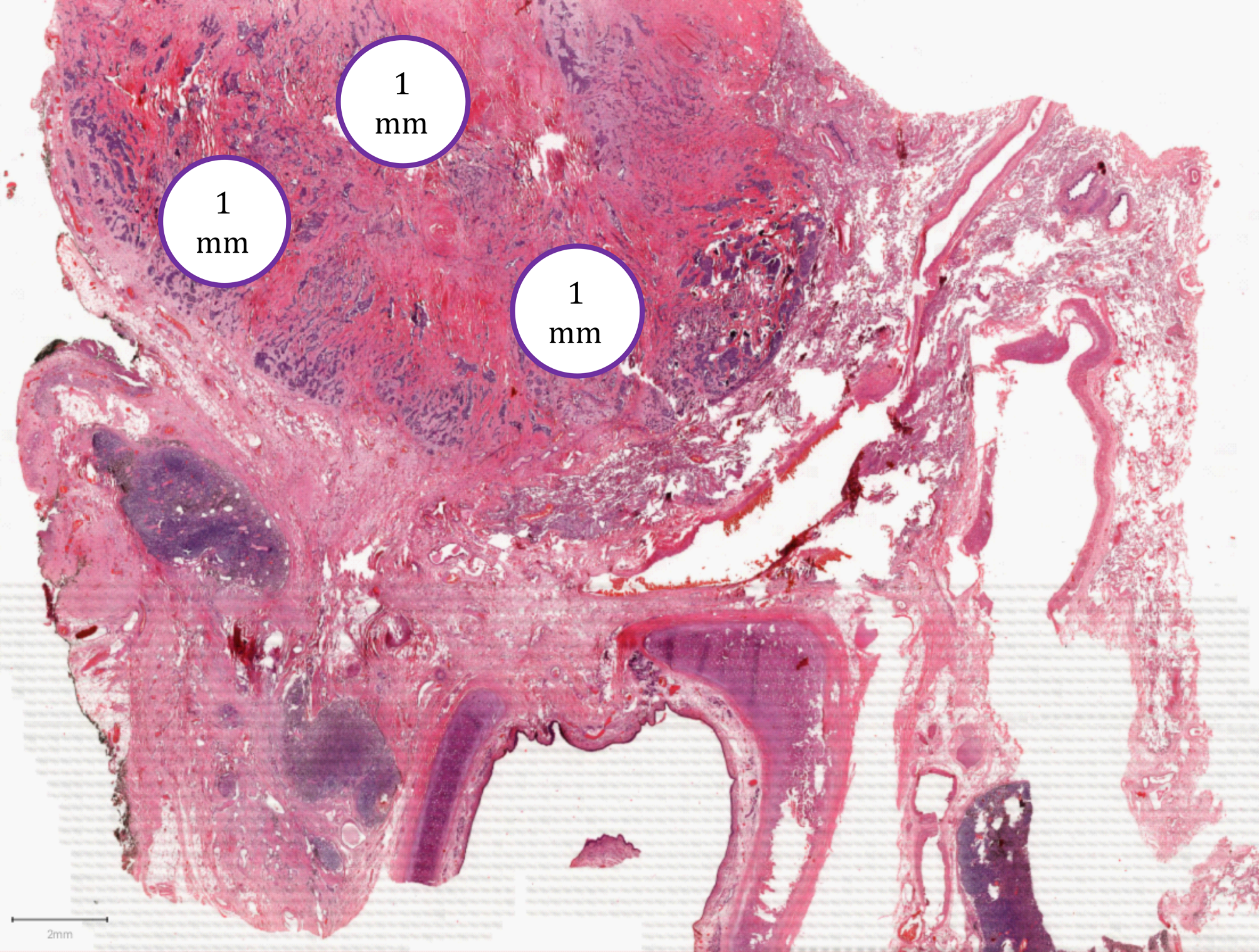

Supplement: Supplementary file 3 [file Image_3.TIFF]

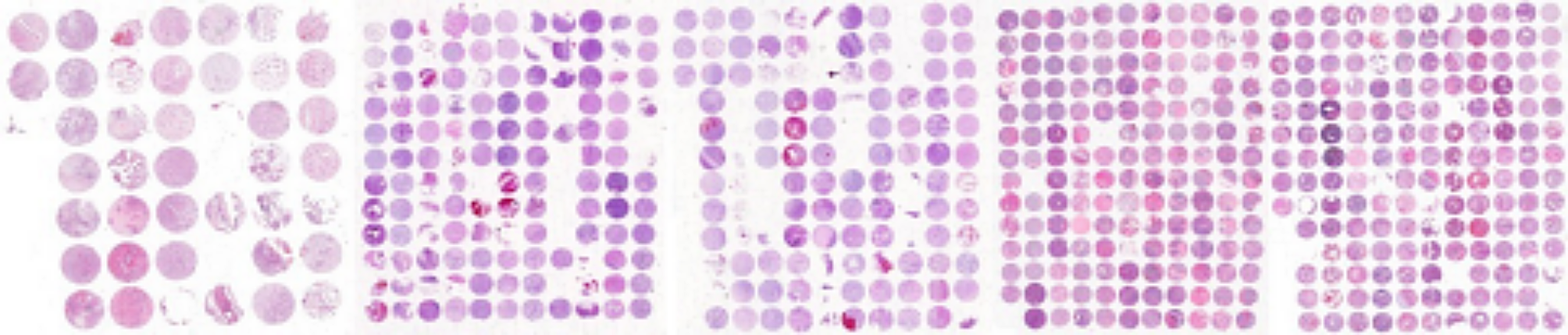

Supplement: Supplementary file 4 [file Image_4.TIFF]

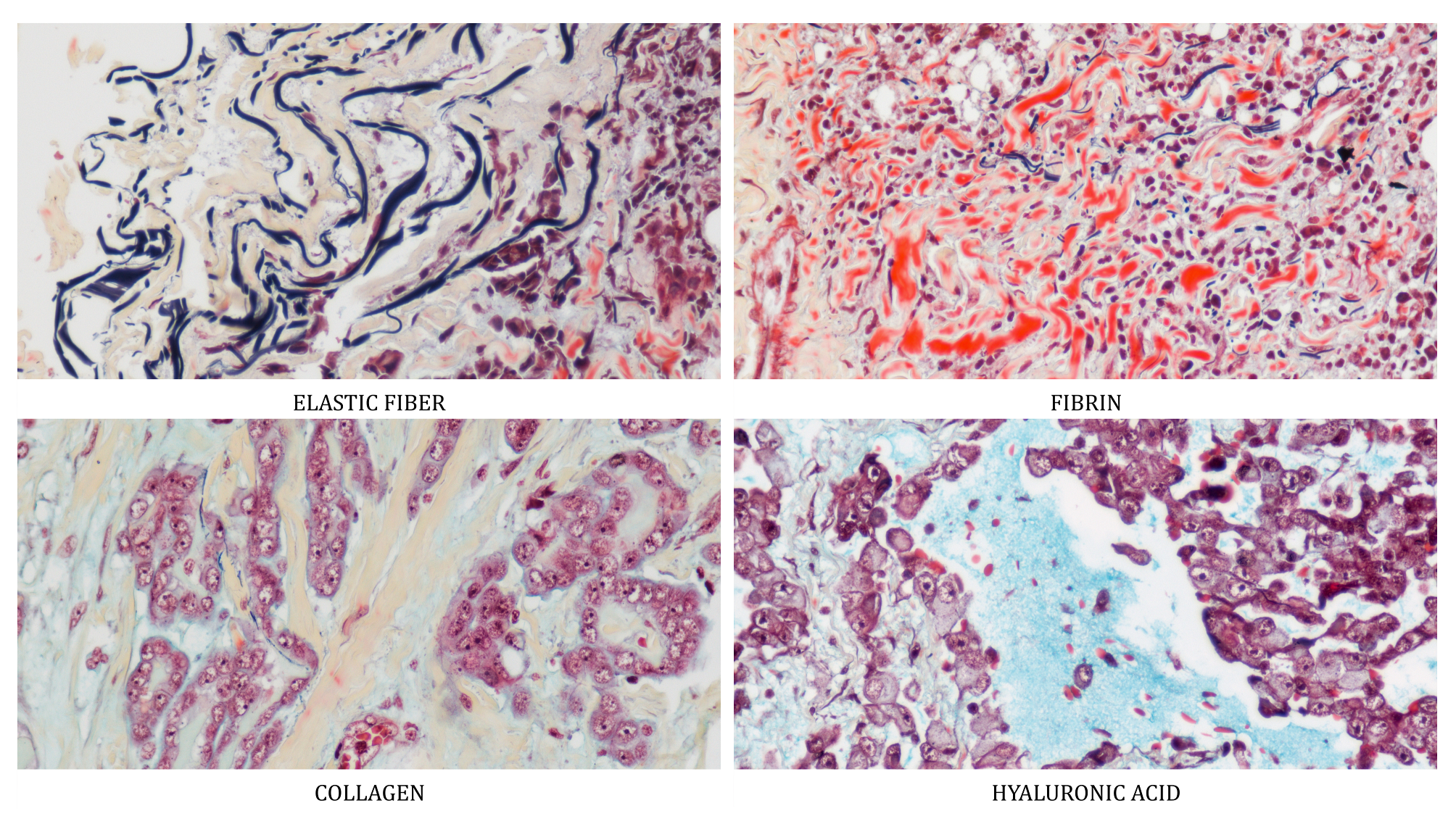

Supplement: Supplementary file 5 [file Image_5.TIFF]

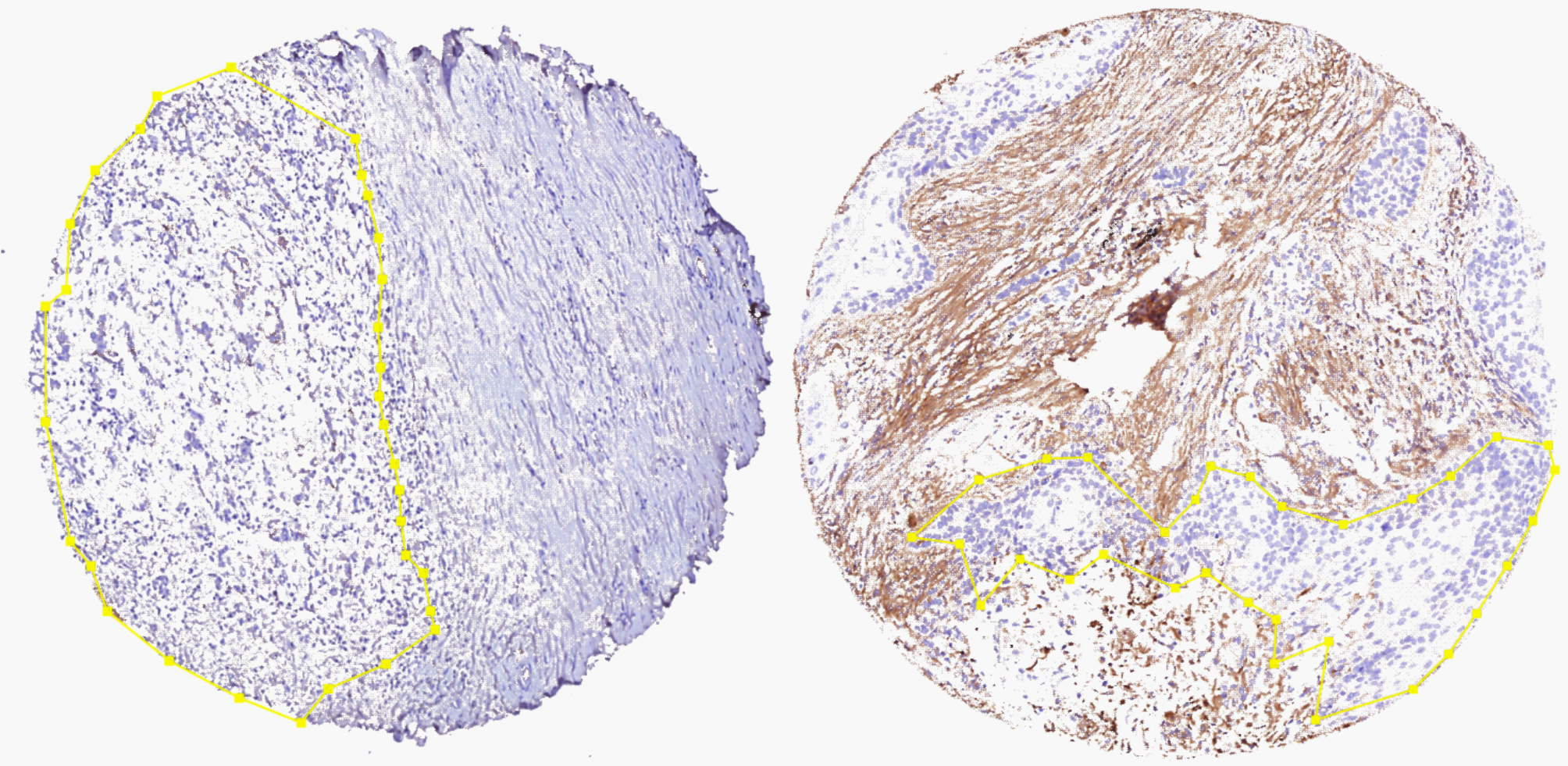

Supplement: Supplementary file 6 [file Image_6.TIFF]

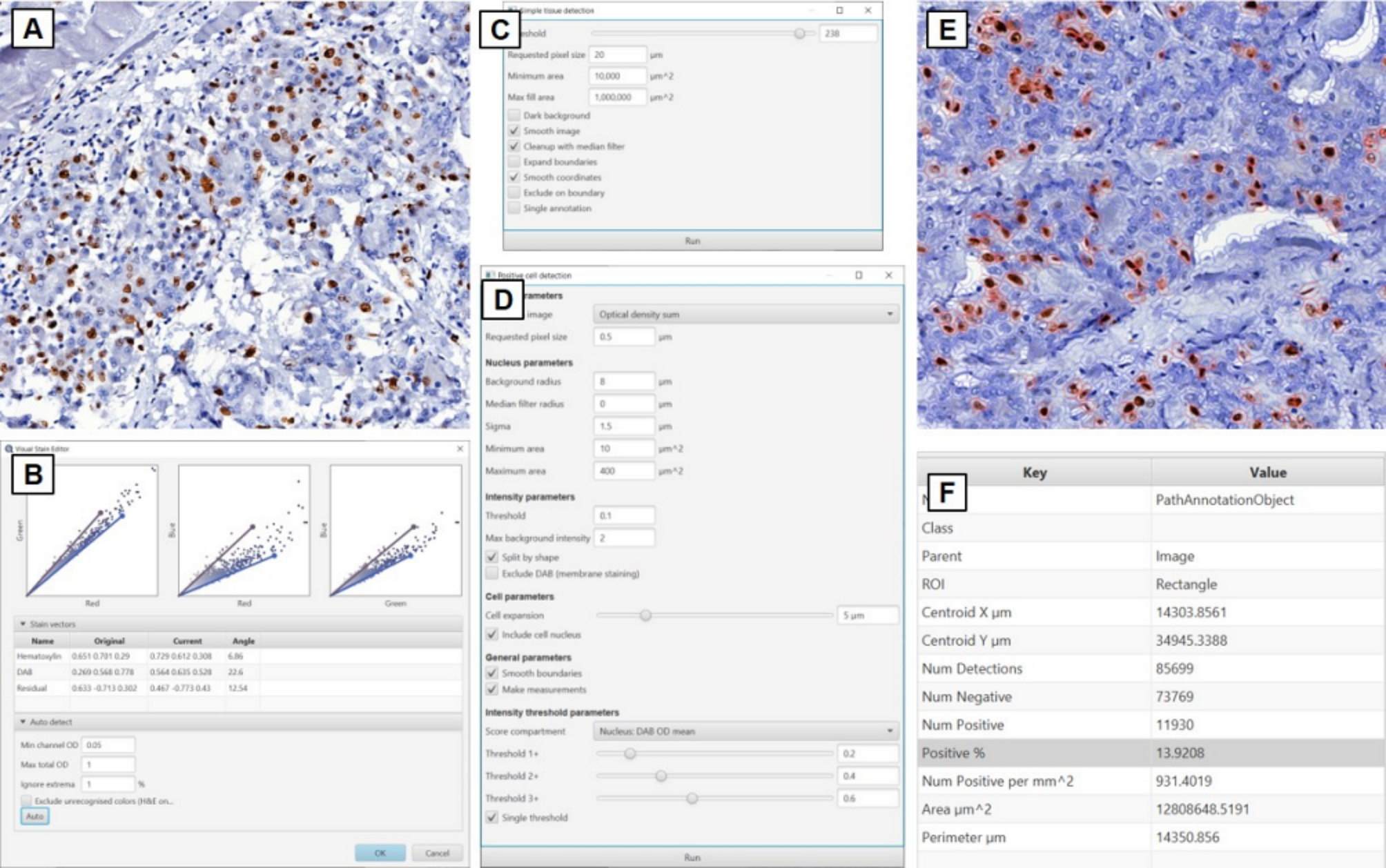

Supplement: Supplementary file 7 [file Image_7.TIFF]

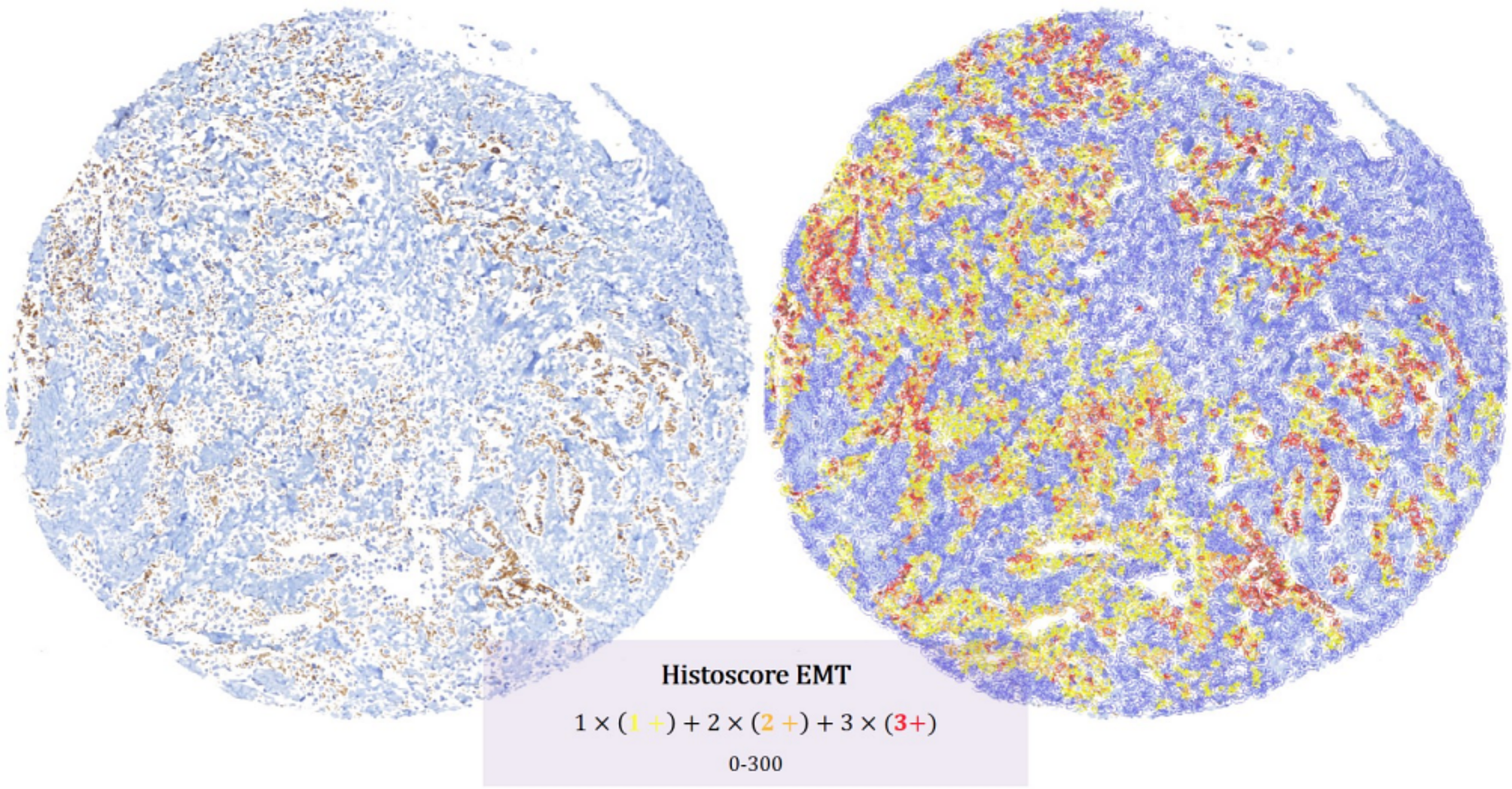

Supplement: Supplementary file 8 [file Image_8.TIFF]

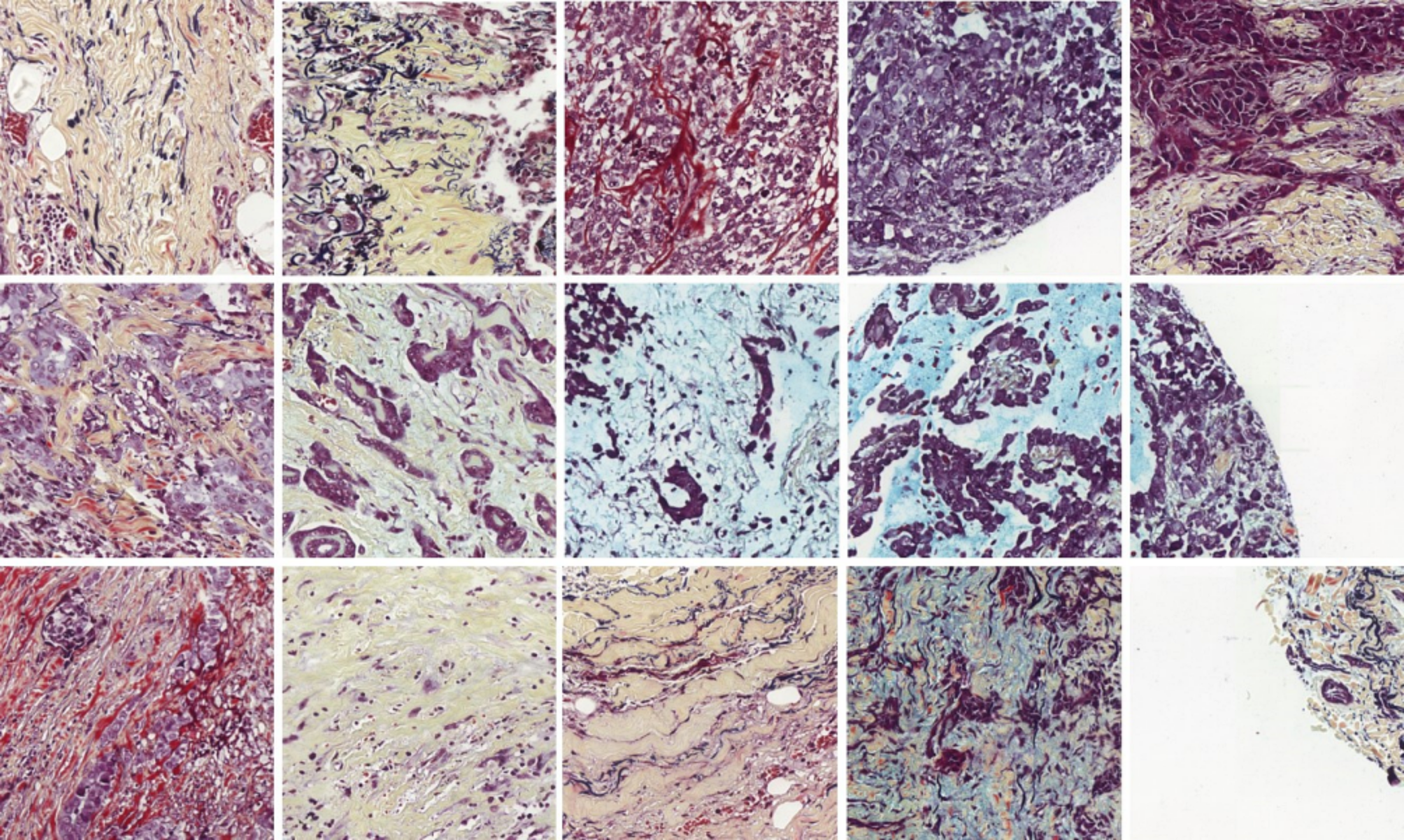

Supplement: Supplementary file 9 [file Image_9.TIFF]

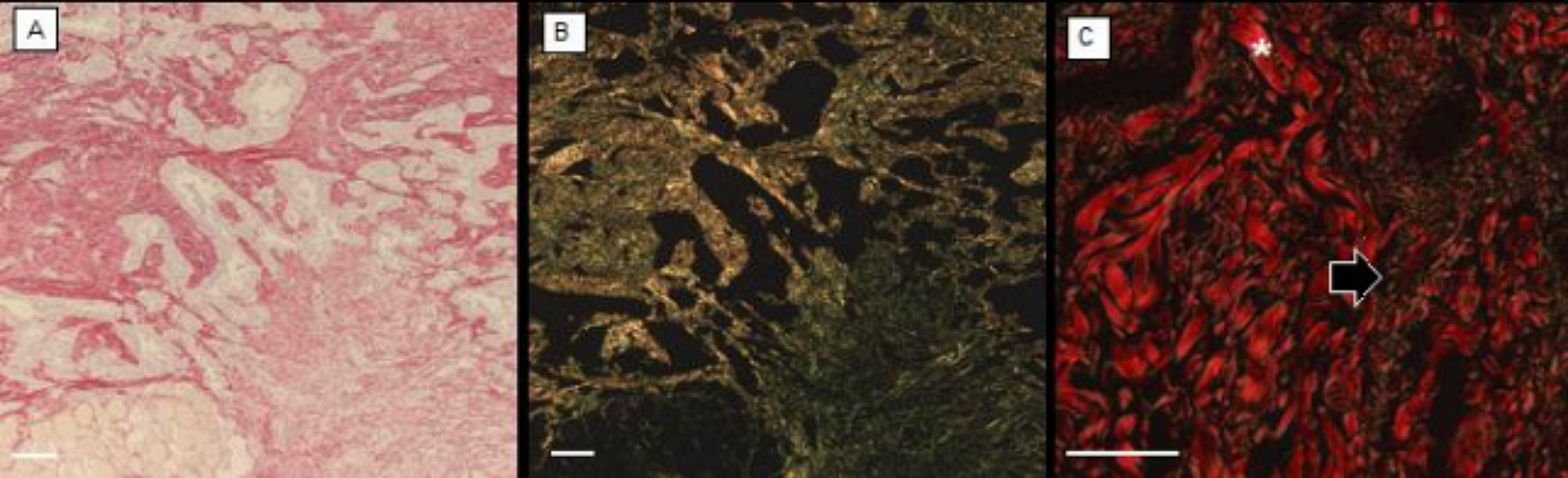

Supplement: Supplementary file 10 [file Image_10.TIFF]

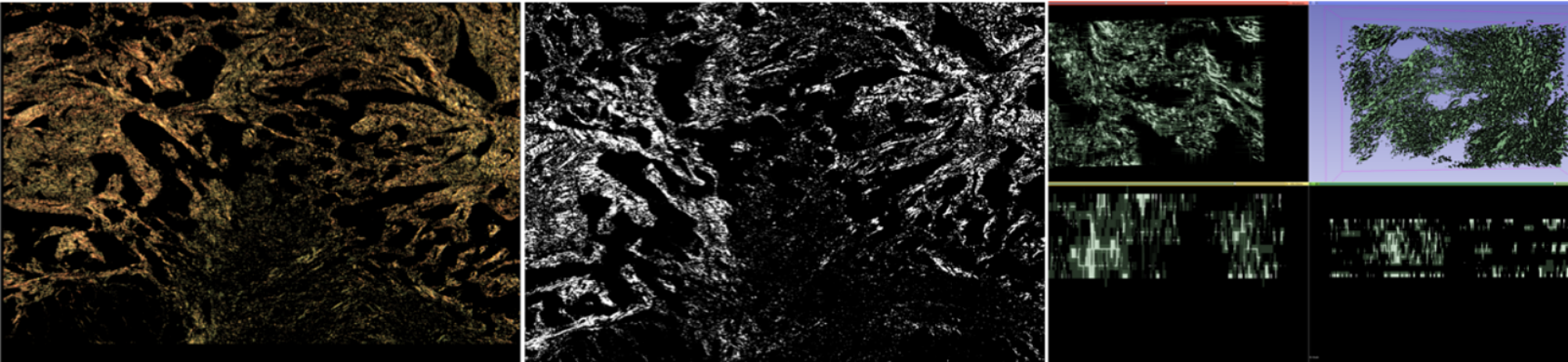

Supplement: Supplementary file 11 [file Image_11.TIFF]

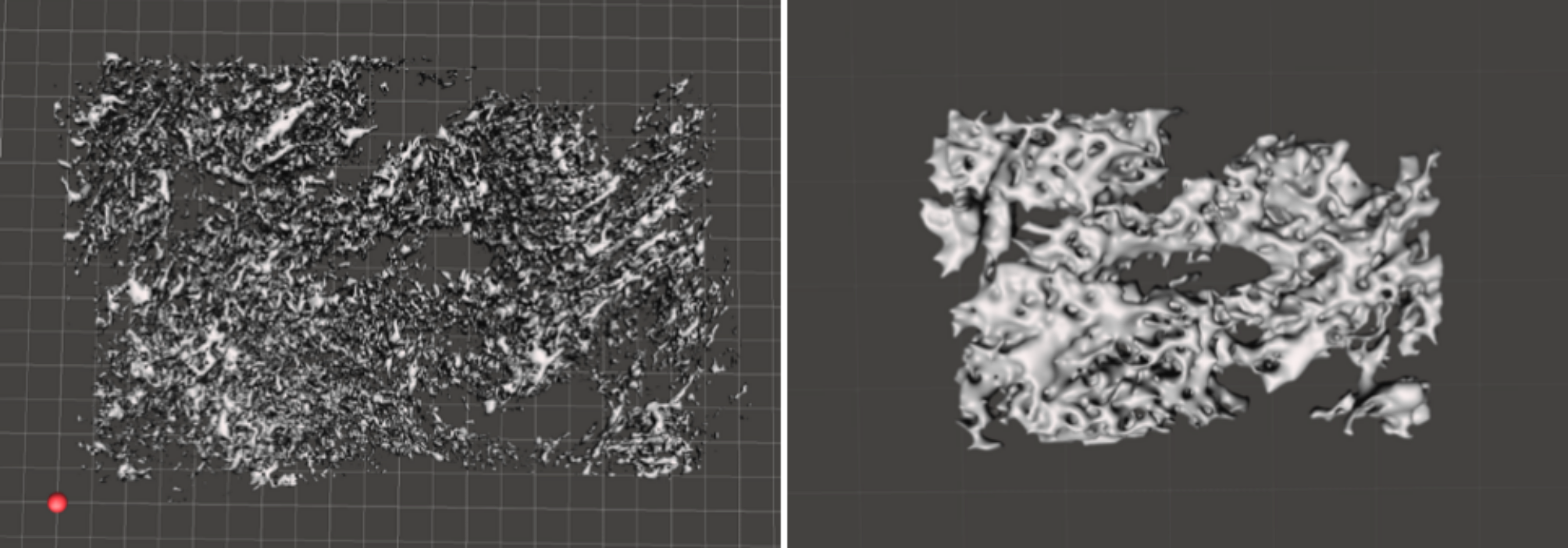

Supplement: Supplementary file 12 [file Image_12.TIF]

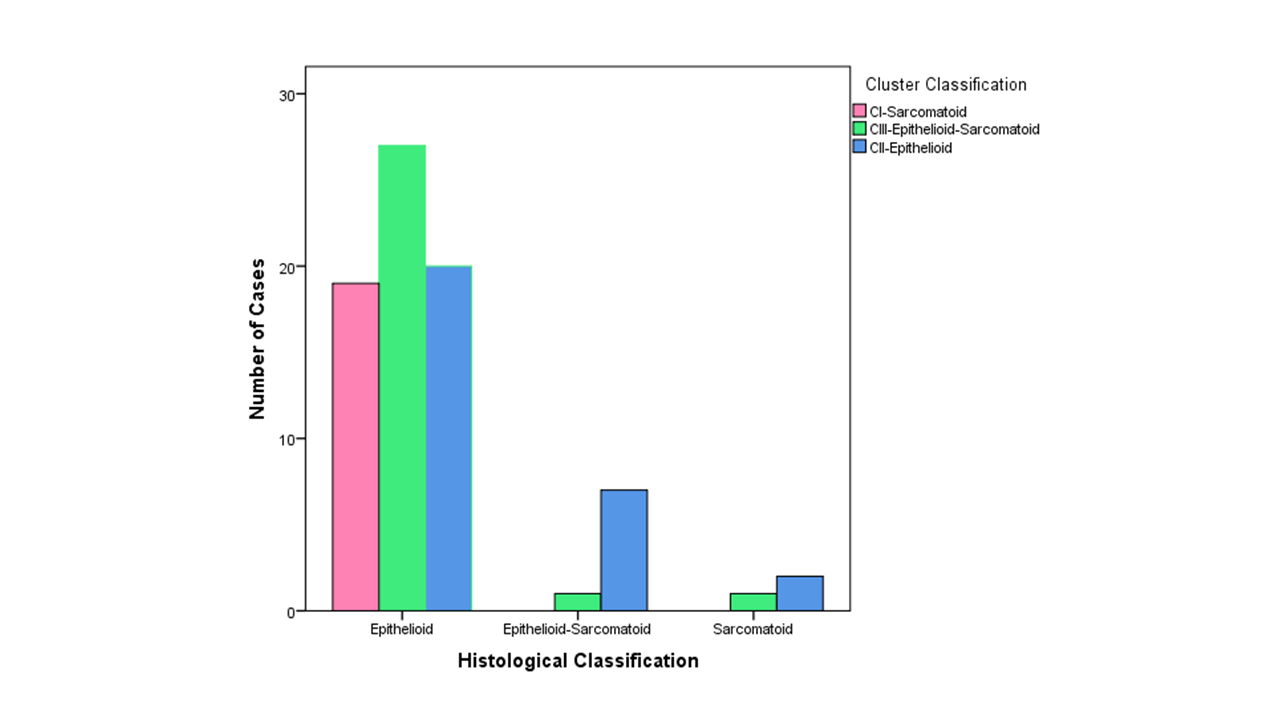

Supplement: Supplementary file 13 [file Image_13.TIF]

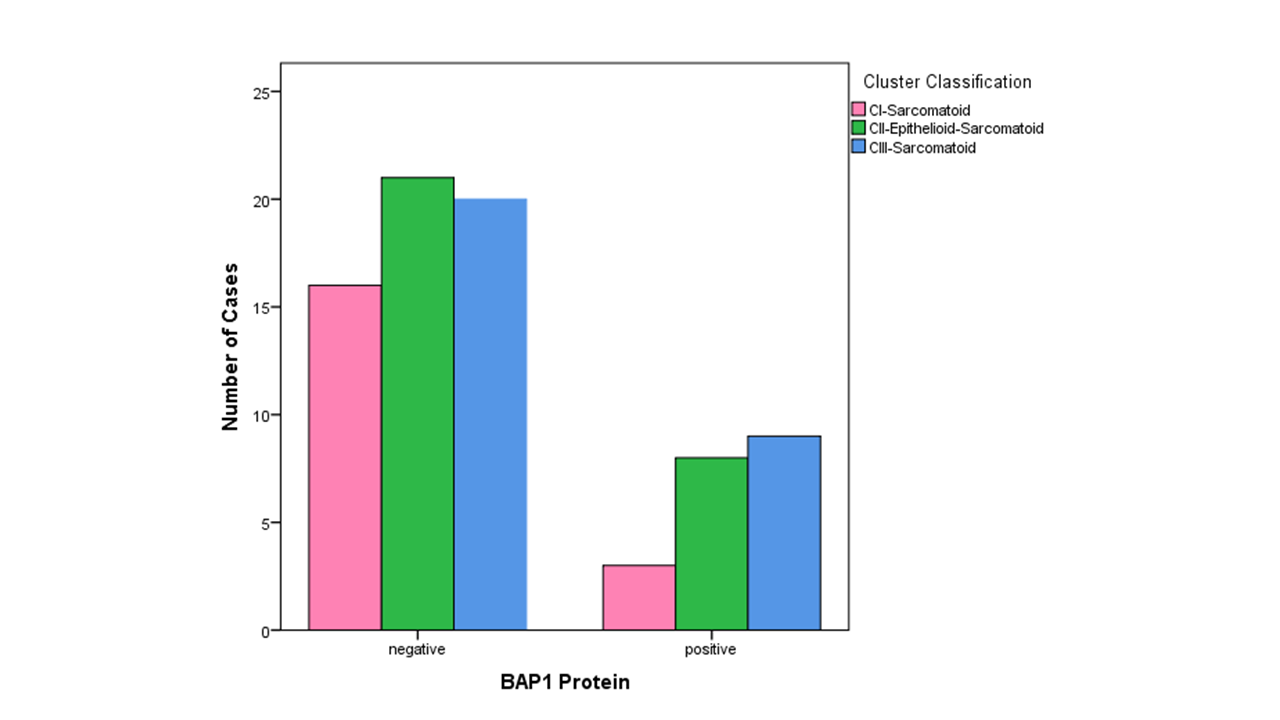

Supplement: Supplementary file 14 [file Image_14.TIF]
