# Supplementary material for: Dissecting and Reconstructing Matrix in Malignant Mesothelioma Through Histocell-Histochemistry Gradients for Clinical Applications
Source: Front Med (Lausanne). 2022 Apr 13;9:871202. doi: 10.3389/fmed.2022.871202 (PMC9043486; doi:10.3389/fmed.2022.871202)
Supplement: Supplementary file 15 [file Data_Sheet_1.pdf]

**Supplementary Table 1**. Connective tissue elements, including muscle tissue and ECM elements, identified by Modified Russel- Movat's pentachrome stain (“Movat's pentachrome”) and their corresponding tones.

| **Connective tissue component** | **Color tone** |
|---|---|
| Elastic fibers | Black to dark blue |
| Collagen/reticulin fibers | Yellow |
| Mucin/hyaluronic acid | Light blue |
| Fibrin | Light red |
| Muscle fibers | Red |

**Supplementary Table 2.** Association between the three clusters classification with the final histotype (epithelioid, biphasic or sarcomatoid) resulting from pathological classification (***$X^2$*; P=0.02**).

| | | **Cluster Classification** | | |
|---|---|---|---|---|
| | | 1 | 2 | 3 |
| **Histological Classification** | Epithelioid | 19 (24.7%) | 27 (35.1%) | 20 (26.0) |
| | Epithelioid-Sarcomatoid | 0 (0.0%) | 1 (1.3%) | 7 (9.1%) |
| | Sarcomatoid | 0 (0.0%) | 1 (1.3%) | 2 (2.6%) |

**Supplementary Table 3.** Stratification of BAP1 protein according to the three clusters (*$X^2$*; P=0.48)

| | | **BAP1 Protein** | |
|---|---|---|---|
| | | **negative** | **positive** |
| **Cluster Classification** | CI- Sarcomatoid | 16 (20.8%) | 3 (3.90%) |
| | CII- Epithelioid-Sarcomatoid | 21 (27.3%) | 8 (10.4%) |
| | CIII- Epithelioid | 20 (26.0%) | 9 (11.7%) |

**Supplementary Figure 1**. The case search flow and inclusion method used in this study.

**Supplementary Figure 2**. Nuclear characteristics, according to Kadota et al. [6]. Mild nuclear atypia, exhibiting homogeneous nuclei and inconspicuous nucleoli (A), a moderate nuclear atypia characterized by an increase in the nucleus cytoplasm ratio and shape, and by variations in dimensions resulting in prominent nucleoli (B), an intense nuclear atypia showing pleomorphism, prominent nucleoli and high nucleo-cytoplasmic ratio (C). Grade 1, showing less than 10% of TIL presence in the tumor cellularity (D), Grade 2, where TIL cells representation ranges between 10% and 50% (E), and Grade 3, with TILs accounting for more than 50 % of cells (F). (A-C, HE, 630x; D-F, HE, 400x).

**Supplementary Figure 3**. Example of selected points in TMA. Note the preference for heterogeneous areas, without evidence of necrosis (HE, 2.5x).

**Supplementary Figure 4**. Microscopic panoramic view of the constructed TMAs (HE, 0.5x).

**Supplementary Figure 5**. Extracellular matrix elements stained with Movat's pentachrome stain. The elastic fibers, shown in a dense fibrillar conformation, are represented in black or dark blue; fibrin, in light red; collagen or areas of fibrosis, in yellow; and hyaluronic acid (also characterized as mucin), in light blue. (Movat's pentachrome stain, 400x).

**Supplementary Figure 6**. Annotation areas in TMA spots to be used for image analysis in QuPath. The yellowish dashed areas were manually constructed to indicate areas of interest. Quantifications were made in these areas, selected in all TMA spots, to ensure specificity.

**Supplementary Figure 7**. Quantification in QuPath. Areas of interest (A) are subjected to color vector normalization (B), cellularity selection (C), optical threshold determination (D), quantification and estimation of cellularity (E), all of which is validated by an observer before results are issued (F) in an automated process.

**Supplementary Figure 8**. Histoscore digital composition (H-SCORE). The digital quantification method adopted attributed different weights to elements at different intensities. The images show the same spot without and with the detection areas, color-coded (yellow 1+,

orange 2+ and red 3+), according to the established thresholds, as determined in the table above.

**Supplementary Figure 9**. Training set for the Weka algorithm, comprising a mosaic of previously selected areas meant to represent “fundamental truths”.

**Supplementary Figure 10**. Illustration of histochemical characteristics stained using the Picrosirius technique. (A) At lower magnification, Col I and III fibers are indirectly identified by reddish or orange color, a polarization signal; (B) under a polarized light, type I fibers are identified in orange tones, whereas type III fibers are greenish. Note that, under polarized light, only collagen structures are highlighted, while others, such as cell blocks, are not visible (dark field areas without visible structures). (C) At higher magnification and under polarized light, both the thick fibers of Col I (asterisk) and the fine greenish fibers of Col III can be more easily identified. (Picrosirius, 40x, 200x and 630x under polarization).

**Supplementary Figure 11.** Workflow to generate a three-dimensional model of the collagen component (fiber types I and III) in the ECM. The workflow included photographs of polarized sections in areas of interest stained with Picrosirius, the alignment and application of optical threshold, and a digital overlay using the Slicer3D software.

**Supplementary Figure 12.** 3D printing models: (A) Vertex reduction and (B) model created in Autodesk Meshmixer. The software created a “.stl” file, reducing the resolution of triangles and excluding isolated points.

**Supplementary Figure 13**. Plots to demonstrate the association between the three clusters classification with the final histotype (epithelioid, biphasic or sarcomatoid) resulting from pathological classification **(($X^2$; P=0.02)**.

**Supplementary Figure 14**. Plots to demonstrate the association between the three clusters classification with the final histotype (epithelioid, biphasic or sarcomatoid) resulting from pathological classification ($X^2$, P=0.48).
